# Genome-wide identification, classification, and characterization of lectin gene superfamily in sweet orange (*Citrus sinensis* L.)

**DOI:** 10.1371/journal.pone.0294233

**Published:** 2023-11-13

**Authors:** Fee Faysal Ahmed, Farah Sumaiya Dola, Fatema Tuz Zohra, Shaikh Mizanur Rahman, Jesmin Naher Konak, Md. Abdur Rauf Sarkar

**Affiliations:** 1 Department of Mathematics, Faculty of Science, Jashore University of Science and Technology, Jashore, Bangladesh; 2 Department of Genetic Engineering and Biotechnology, Faculty of Biological Science and Technology, Jashore University of Science and Technology, Jashore, Bangladesh; 3 Department of Genetic Engineering and Biotechnology, Faculty of Biological Sciences, University of Rajshahi, Rajshahi, Bangladesh; 4 Department of Biochemistry and Molecular Biology, Faculty of LifeScience, Mawlana Bhashani Science and Technology University, Santosh, Tangail, Bangladesh; Agri Biotech Foundation and Retired Professor, University of Hyderabad, INDIA

## Abstract

Lectins are sugar-binding proteins found abundantly in plants. Lectin superfamily members have diverse roles, including plant growth, development, cellular processes, stress responses, and defense against microbes. However, the genome-wide identification and functional analysis of lectin genes in sweet orange (*Citrus sinensis* L.) remain unexplored. Therefore, we used integrated bioinformatics approaches (IBA) for in-depth genome-wide identification, characterization, and regulatory factor analysis of sweet orange lectin genes. Through genome-wide comparative analysis, we identified a total of 141 lectin genes distributed across 10 distinct gene families such as 68 CsB-Lectin, 13 CsLysin Motif (LysM), 4 CsChitin-Bind1, 1 CsLec-C, 3 CsGal-B, 1 CsCalreticulin, 3 CsJacalin, 13 CsPhloem, 11 CsGal-Lec, and 24 CsLectinlegB.This classification relied on characteristic domain and phylogenetic analysis, showing significant homology with *Arabidopsis thaliana*’s lectin gene families. A thorough analysis unveiled common similarities within specific groups and notable variations across different protein groups. Gene Ontology (GO) enrichment analysis highlighted the predicted genes’ roles in diverse cellular components, metabolic processes, and stress-related regulation. Additionally, network analysis of lectin genes with transcription factors (TFs) identified pivotal regulators like ERF, MYB, NAC, WRKY, bHLH, bZIP, and TCP. The cis-acting regulatory elements (CAREs) found in sweet orange lectin genes showed their roles in crucial pathways, including light-responsive (LR), stress-responsive (SR), hormone-responsive (HR), and more. These findings will aid in the in-depth molecular examination of these potential genes and their regulatory elements, contributing to targeted enhancements of sweet orange species in breeding programs.

## 1.0 Introduction

Lectins, a unique class of carbohydrate-binding proteins, play various roles in plants. Lectins, also known as glycan-binding proteins, selectively attach to specific carbohydrates or carbohydrate-containing biomolecules (glycoconjugates) without altering their structures, and this binding is reversible [1–3]. Plants are the primary source of lectins, but they are also found in humans and viruses [4]. The initial plant lectin was identified in castor bean (*Ricinus communis* L.) seeds [5]. Typically, plant lectins are distributed throughout all plant organs, with higher concentrations found in seeds, bulbs, bark, rhizomes, and corns, and lower amounts in shoots, leaves, roots, and flowers. Plant lectins are pivotal in essential developmental processes and serve as a crucial part of plant immune and defense systems, responding to both biotic and abiotic stresses. These lectin proteins exhibit variations in molecular structure and specificity across different plant species [6]. Lectins are grouped into three categories based on their cellular location: membrane lectins (in organelles and cytoplasmic membranes), soluble lectins (in vacuolar sap and cytosol), and cell wall lectins. Membrane and soluble lectins enhance resistance to salinity, while cell wall lectins are linked to resistance against low temperatures [7–12].

In addition, based on the number of carbohydrate-binding domains, there are three major types of lectins: merolectins, hololectins, and chimerolectins. Merolectins have a single carbohydrate-binding domain and are monovalent, incapable of regulating glycoconjugates. In contrast, hololectins possess two or more identical or highly homologous domains, allowing them to down-regulate glycoconjugates or clump cells. This group encompasses most plant lectins. Chimerolectins are fusion proteins that combine one or more carbohydrate-binding domains with unrelated domains [6]. A discreet classification of seven families has been proposed based on sequence similarities, serological relationships, and evolutionary connections. These families comprise legume lectins, chitin-binding lectins, monocot-mannose binding lectins, jacalin-related lectins, type-2 RIP and related lectins, cucurbitaceae phloem lectins, and amaranthin lectins [12].

Numerous plant lectins exhibit promising antibacterial, antifungal, and antiviral properties. Specifically, plant-derived lectins like SLL-1, SLL-2, and SLL-3 significantly inhibited the growth of *Escherichia coli*, *Shigella dysenteriae*, and *Staphylococcus aureus* [13]. Chitin-binding lectins (CBLs) from *Solanum integrifolium* exhibited immune-defense-like hemagglutination activity (HA) against chitin-containing pathogens *Rhizoctonia solani* and *Colletotrichum gloeosporioide*, inhibiting their growth [14]. Previous studies have shown that lectins isolated from Egyptian *P. sativum* seeds possess antifungal activity against pathogens such as *Aspergillus flavus*, *Fusarium oxysporum*, and *Trichoderma viride* [15]. Plant-derived lectins have insecticidal properties against a range of insect species. For instance, ASAL, a GNA-related lectin from garlic, has demonstrated toxicity against Hemiptera, sucking aphids [16, 17]. Plant-derived lectins have recently captivated researchers due to their multifaceted potential in human health. They are seen as innovative tools in technology for diagnosing and treating significant diseases [5]. Certain plant-based lectins exhibit mitogenic effects on human cells, triggering apoptotic or autophagic processes. For instance, a lectin from *Morus alba* leaves induced apoptosis and increased caspase-3 activity in the MCF-7 human breast cancer cell line [18]. Additionally, a lectin from *Bauhinia forficata* seeds accelerated necrosis and inhibited caspase-9 in the MCF-7 human breast cancer cell line [19]. Lectin-based microarray technology has emerged as a crucial biomarker for high-throughput, stable, rapid, and sensitive analysis, making it an important diagnostic tool for severe human diseases [20]. Plant lectins play a key role in agglutinating and immobilizing *Rhizobium* or *Bradyrhizobium* bacteria. Under various abiotic stresses like temperature shock, high salinity, and drought, certain lectin genes show altered expression levels [7, 21–25].

Sweet orange (*Citrus sinensis* L.), part of the Rutaceae family, is a globally significant fruit crop, prized for its essential nutrient composition. It serves as a primary raw material in the food, pharmaceutical, and cosmetic industries. Notably, sweet orange contributes to around 70% of the total annual citrus fruit production worldwide. Sweet orange is a valuable fruit known for its rich content of natural antioxidants, vitamin C, flavonoids, minerals (sodium, calcium, potassium, magnesium), steroids, fatty acids, and alkanes [26–29]. Its antioxidants and nutrients find applications in treating various conditions, including digestive issues, immune enhancement, and anxiety relief. Additionally, numerous plant lectins have been recently discovered, showing diverse biological activities such as antitumor effects, immune modulation, and antiviral activity [30–33]. Citrus fruit species often face environmental challenges like abiotic and biotic stresses, which can reduce yield and quality by affecting growth and development [34]. To sustain the citrus industry’s raw material supply, it’s vital to comprehend gene expression, metabolite analysis, and breed stress-resistant citrus cultivars. This strategy is crucial to meet the demands of the competitive global citrus market [35].

**Fig 1:**
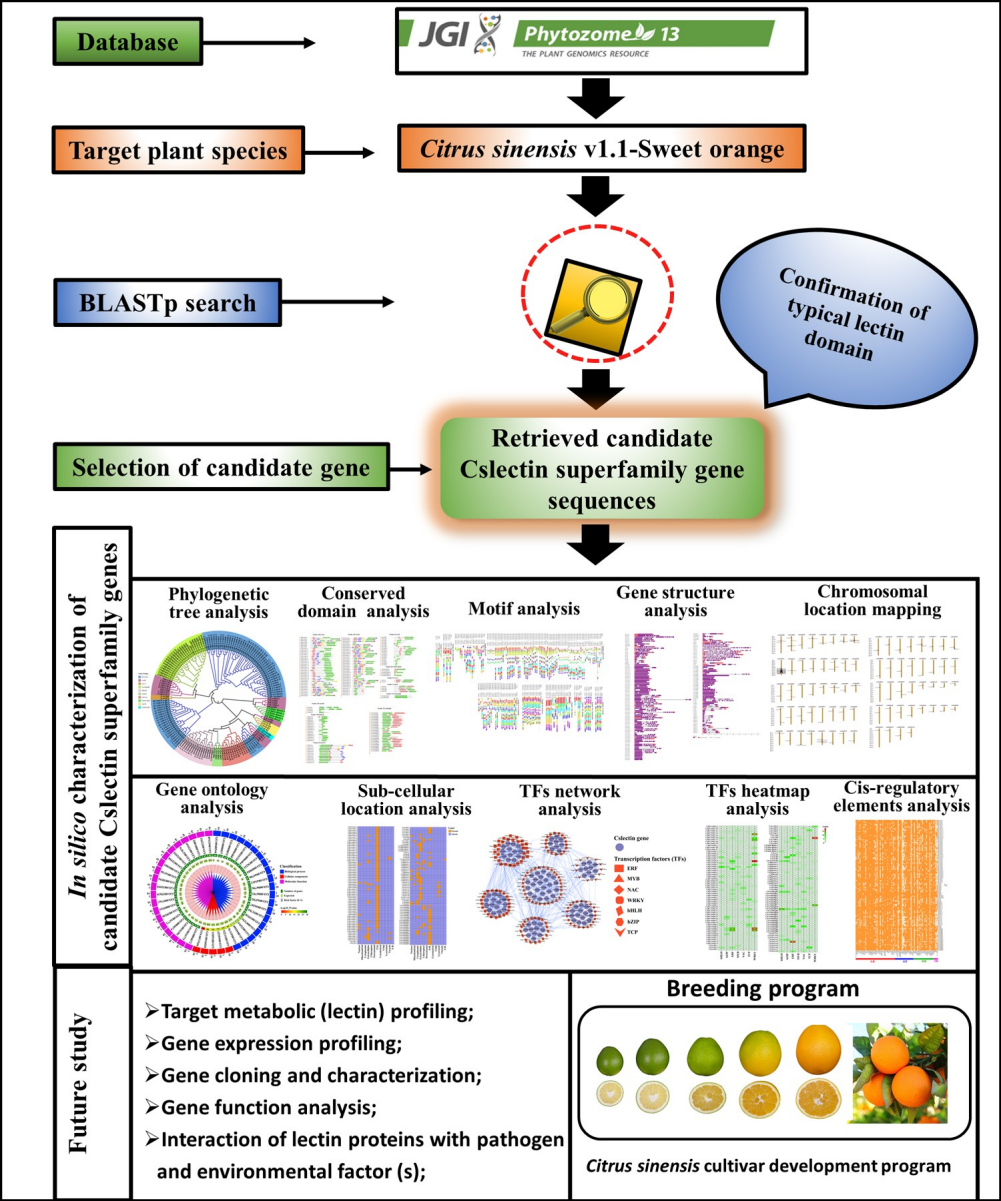
The overview strategy of our present study.

In this study, we aimed to identify and characterize the lectin gene superfamily in the *C. sinensis* genome using bioinformatics approaches. Sweet orange lectins have potential in agriculture and human health. Modifying lectin gene expression and introducing new ones through biotechnology can enhance its growth and quality. Our findings provide insights for wet-lab investigations and for improving sweet oranges in breeding programs. We have schematically represented our approach for this study in Fig 1.

## 2.0 Materials and methods

### 2.1 Identification of lectin gene superfamily in sweet orange (*C. sinensis)* genome

The complete genome data and protein sequences of sweet orange (*C. sinensis)* were obtained from Phytozome v13.0 (https://phytozome-next.jgi.doe.gov/). To retrieve the all members of the lectin genes in the *C. sinensis* genome, we collected the published lectin gene family sequence and annotation information of Arabidopsis from the TAIR (http://www.arabidopsis.org/) database. The lectin family protein domains were confirmed from the Pfam database (http://pfam.xfam.org/) by using the Hidden Markov Model (HMM) profile and then the typical lectin protein domains were used to search for sweet orange lectin genes with the Basic Local Alignment Search Tool (BLAST) program. We obtained sweet orange genome data and protein sequences from Phytozome v13.0 (https://phytozome-next.jgi.doe.gov/). To identify lectin genes, we collected lectin gene family sequences and annotations from Arabidopsis in the TAIR database (http://www.arabidopsis.org/). We confirmed lectin protein domains using the Pfam database (http://pfam.xfam.org/) with Hidden Markov Model profiles and used these domains to search for sweet orange lectin genes through the BLAST program. We downloaded candidate sweet orange lectin protein sequences with a minimum identity of 30% (BLOSUM62 matrix) and significant E-values (≤10E-10) from Phytozome v13.0. We further identified possible lectin family sequences in sweet orange using Pfam’s online tools for predicting conserved protein domains. The coding sequences, genomic sequences, and protein sequences of candidate lectin gene family members of sweet orange are presented in S1–S3 Data. We retrieved primary transcripts, gene lengths, chromosomal locations, and open reading frames (ORFs) of these lectin genes from the sweet orange genome database in Phytozome. We assessed the physiochemical properties of sweet orange lectin proteins, including length, molecular weight, isoelectric points (pI), and average hydrophilicity (GRAVY), using online ExPASy tools (https://web.expasy.org/protparam/).

### 2.2 Phylogenetic relationship of lectin proteins in sweet orange and Arabidopsis

We used MEGA 11.0 software [36] to construct a phylogenetic tree from lectin protein sequences in sweet orange and Arabidopsis. We aligned the sequences using Clustal-W [37] with default parameters and 1000 bootstrap values. The tree was built using the Neighbor-joining method [38] via Clustal Omega (https://www.ebi.ac.uk/Tools/msa/clustalo/), and evolutionary distances were calculated using the Equal Input method [39].

### 2.3 Domain and motif analysis of lectin proteins in sweet orange

We analyzed conserved domains in sweet orange lectin genes alongside Arabidopsis lectin genes using the Pfam database (http://pfam.xfam.org/) with significant E-values (≤10E-10). Additionally, we predicted structural motif similarities and differences between sweet orange and Arabidopsis proteins using the Multiple Expectation Maximization for Motif Elicitation (MEME-Suite v5.5.3) server (https://meme-suite.org/meme/tools/meme) [40] with significant *p*-values (p-val<0.05). The MEME analysis used specific parameters: (i) optimal motif width between ≥6 and ≤50 and (ii) a maximum of 20 motifs.

### 2.4 Analysis of gene structure and chromosomal location of lectin proteins in sweet orange

We analyzed the predicted lectin gene structures (exon-intron) using the online software Gene Structure Display Server GSDS2.0 (http://gsds.gao-lab.org/) [41]. This analysis was based on DNA sequences of the identified lectin genes compared with Arabidopsis lectin genes. Furthermore, we determined the chromosomal locations of the lectin gene family in sweet orange using MapGene2Chromosome V2 (http://mg2c.iask.in/mg2c_v2.0/) [42].

### 2.5 Analysis of gene ontology and sub-cellular localization of lectin proteins in sweet orange

Gene ontology (GO) analysis was carried out to predict the relationship of identified lectin genes with the group of various biological processes and molecular functional pathways using an online tool called Plant Transcription Factor Database (PlantTFDB 4.0), (http://planttfdb.gao-lab.org/) [43] with the significant *p*-values (*p*-val<0.05). The sub-cellular location of the identified lectin proteins was predicted in the various cell organelles by an online predictor named Plant Subcellular Localization Integrative Predictor (PSI) (https://bis.zju.edu.cn/psi/) [44].

### 2.6 Regulatory relationship between transcription factors and lectin genes in sweet orange

Important TFs associated with the identified lectin genes were predicted from PlantTFDB 4.0 (http://planttfdb.gao-lab.org/) [43]. Moreover, lectin genes versus predicted TFs regulatory network were constructed and displayed by Cytoscape 3.9.1 [45].

### 2.7 Analysis of *cis*-acting regulatory elements (CAREs) of lectin proteins in sweet orange

We predicted cis-acting regulatory elements (CAREs) linked to stress responses in the 1.5 kb upstream regions of the identified lectin genes using the Signal Scan search program in the PlantCARE database (https://bioinformatics.psb.ugent.be/webtools/plantcare/html/) [46]. CAREs were categorized into five classes: light-responsive (LR), stress-responsive (SR), hormone-responsive (HR), other activities (OT), and unknown functions based on their regulatory roles.

## 3.0 Results and discussion

### 3.1 Identification of lectin gene family in sweet orange (*C. sinensis*) genome

We used 12 *Arabidopsis thaliana* lectin families (AtB-Lectin, AtLysM, AtChitin-Bind1, AtLec-C, AtGal-B, AtCalreticulin, AtJacalin, AtPhloem, AtGal-Lec, AtLectinlegB, AtRicin-B-Lectin, and AtEEA) as query sequences to identify lectin genes in the sweet orange genome. Using HMM profile analysis, we found 10 lectin superfamilies (CsB-Lectin, CsLysM, CsChitin-Bind1, CsLec-C, CsGal-B, CsCalreticulin, CsJacalin, CsPhloem, CsGal-Lec, and CsLectinlegB) in the sweet orange genome. We identified 141 lectin genes in sweet orange, categorized as follows: 68 genes encoding B-Lectin proteins (CsB-Lectin), 13 genes encoding LysM proteins (CsLysM), 4 genes encoding Chitin-Bind1 proteins (CsChitin-Bind1), one gene encoding Lec-C protein (CsLec-C), three genes encoding Gal-B proteins (CsGal-B), one gene encoding Calreticulin protein (CsCalreticulin), 3 genes encoding Jacalin proteins (CsJacalin), 13 genes encoding Phloem proteins (CsPhloem), 11 genes encoding Gal-Lec proteins (CsGal-Lec) and 24 genes encoding LectinlegB proteins (CsLectinlegB). However, two gene families, Ricin-B-Lectin and EEA, were not found in *C. sinensis* when compared with Arabidopsis. Table 1 presents the identified lectin gene superfamily in sweet orange alongside those previously identified in soybean, rice, and Arabidopsis [47].

**Table 1:** Genome-wide identification of lectin gene superfamily in sweet orange (*C. sinensis*) and comparison with previously identified soybean, rice, and Arabidopsis plant species [47].

				Species	
SI	Family	*Citrus sinensis*	*Glycine max*	*Oryza sativa*	*Arabidopsis thaliana*
1	B-Lectin	68	105	87	38
2	LysM	13	34	16	9
3	Chitin-Bind1	4	7	10	8
4	Lec-C	1	2	1	1
5	Gal-B	3	15	10	6
6	Calreticulin	1	10	6	5
7	Jacalin	3	6	28	46
8	Phloem	13	28	20	30
9	Gal-Lec	11	33	12	13
10	LectinlegB	24	66	69	41
11	Ricin-B-Lectin	0	1	1	1
12	EEA	0	2	7	1
	**Total**	**141**	**309**	**267**	**199**

Moreover, Table 2 showcases the identified lectin genes, their genomic positions, ORF length, isoelectric points (pI), protein length, and molecular weight. The amino acid sequences of the 68 CsB-Lectin genes contained typical conserved domains of the plant B-Lectin family, including Pkinase, B_lectin, Pk_Tyr_Ser_Thr, S locus glycop, PAN 2, and DUF3403. The CsB-Lectin genes had ORF lengths ranging from 348bp (CsB-Lectin6.13) to 3057bp (CsB-Lectin2.4), encoding amino acid sequences of 116 and 1019 aa, respectively. The pI values of these CsB-Lectin proteins varied, with CsB-Lectin5.1 having the highest pI of 8.60 (basic) and CsB-Lectin4.7 having the lowest pI of 4.96 (acidic). Additionally, 13 CsLysM genes contained typical domains such as LysM, Pkinase, and PK_Tyr_Ser_Thr, placing them within the plant LysM protein family. We found that CsLysMs ORF ranged from 315 to 2373 bp, corresponding to CsLysM6.3 and CsLysM1.2, which encoded proteins of 105 and 791 aa, respectively (Table 2). The pI values of CsLysMs ranged from 4.97 (acidic) in CsLysM2.2 to 9.28 (basic) in CsLysM3.2.The significant conserved domains, Glyco hydro 19 and Chitin_bind_1, were predicted in the peptide sequence of the CsChitin-Bind1 gene to validate its inclusion in the plant Chitin-Bind1 family. The determined ORFs of CsChitin-Bind1 ranged from 237 bp to 990 bp, corresponding to CsChitin-Bind1.3 and CsChitin-Bind1.2. Based on the pI values of the CsChitin-Bind1 proteins, CsChitin-Bind1.1 and CsChitin-Bind1.4 exhibited acidic properties, while CsChitin-Bind1.2 and CsChitin-Bind1.3 displayed basic properties. The Lec-C family in sweet orange was observed to contain Pk_Tyr_Ser_Thr domain along with the Lectin_C domain. The ORF length of the predicted CsLec-C was 1722 bp with the encoded protein length 574 aa and the pI value (9.04) of CsLec-C demonstrating higher basic properties. Our HMM analysis predicted the Gal_bind_lectin and Galactosyl_T conserved domain in the CsGal-B gene family. The identified ORF length ranged from 1887 bp to 1992 bp, indicating CsGal-B2 and CsGal-B3, potentially encoding amino acids 629 and 664 aa, respectively.CsGal-B1 and CsGal-B3 were found to have acidic properties, while CsGal-B2 had the highest pI value of 7.61, indicating basic properties.

**Table 2:** Basic information of lectin gene superfamily of *C. sinensis*.

SI	Name	Gene ID	Chromosome number	Genomic location	ORF (bp)	Introns number	Protein		
							Molecular weight (kD)	Protein length (aa)	pI value
1	CsB-Lectin1.1	orange1.1g036583m	1	478637..481091	2454	0	89.49062	818	6.91
2	CsB-Lectin1.2	orange1.1g046493m	1	4397..6434	2037	0	75.91454	679	7.14
3	CsB-Lectin1.3	orange1.1g037563m	1	98464..100497	1818	1	67.63448	606	7.73
4	CsB-Lectin1.4	orange1.1g043185m	1	806593..808765	2172	0	79.40915	724	6.17
5	CsB-Lectin1.5	orange1.1g003059m	1	1511007..1513751	2559	0	92.3765	853	7.75
6	CsB-Lectin1.6	orange1.1g038743m	1	299696..303244	2520	4	92.95306	840	5.81
7	CsB-Lectin1.7	orange1.1g044374m	1	2117568..2120154	2415	1	89.34857	805	6.45
8	CsB-Lectin1.8	orange1.1g046586m	1	368410..370810	2292	1	85.92802	764	7.09
9	CsB-Lectin1.9	orange1.1g038103m	1	120263..122678	2415	0	90.60706	805	7.92
10	CsB-Lectin1.10	orange1.1g038002m	1	61104..64675	2109	1	78.13587	703	7.96
11	CsB-Lectin1.11	orange1.1g003772m	1	83007..85495	2391	0	89.68554	797	6.17
12	CsB-Lectin1.12	orange1.1g003818m	1	369456..372024	2382	0	88.75065	794	5.31
13	CsB-Lectin1.13	orange1.1g040134m	1	150235..152473	2238	0	84.55009	746	6.21
14	CsB-Lectin1.14	orange1.1g044281m	1	22349..24740	2391	0	89.4217	797	6.43
15	CsB-Lectin1.15	orange1.1g047292m	1	11941..14128	2187	0	82.02524	729	5.91
16	CsB-Lectin1.16	orange1.1g038294m	1	30408..32808	2400	0	89.89049	800	7.07
17	CsB-Lectin1.17	orange1.1g047263m	1	356426..358841	2415	0	89.94427	805	5.42
18	CsB-Lectin1.18	orange1.1g003836m	1	359716..362501	2379	0	89.13973	793	6.22
19	CsB-Lectin1.19	orange1.1g042262m	1	286136..288581	2445	0	91.51835	815	5.2
20	CsB-Lectin1.20	orange1.1g041921m	1	320118..322563	2445	0	91.46972	815	6.18
21	CsB-Lectin1.21	orange1.1g045343m	1	251560..254002	2442	0	91.3075	814	5.56
22	CsB-Lectin2.1	orange1.1g036207m	2	63844..68198	2352	3	87.47761	784	5.48
23	CsB-Lectin2.2	orange1.1g005803m	2	15338..19421	2031	5	76.26752	677	8.53
24	CsB-Lectin2.3	orange1.1g043278m	2	24565..27602	2259	6	84.69635	753	5.99
25	CsB-Lectin2.4	orange1.1g046791m	2	12336..21583	3057	10	11.435314	1019	7.15
26	CsB-Lectin3.1	orange1.1g003489m	3	28137..31632	2451	7	91.86438	817	6.43
27	CsB-Lectin3.2	orange1.1g003599m	3	261808..265562	2427	7	91.75437	809	6.13
28	CsB-Lectin3.3	orange1.1g003045m	3	1393006..1397528	2565	6	94.9852	855	6.08
29	CsB-Lectin4.1	orange1.1g003832m	4	1638979..1642167	2379	6	88.93159	793	6.95
30	CsB-Lectin4.2	orange1.1g044879m	4	105..3124	1959	5	73.583	653	8.09
31	CsB-Lectin4.3	orange1.1g003254m	4	1656451..1660163	2511	6	94.03529	837	8.25
32	CsB-Lectin4.4	orange1.1g003272m	4	1692259..1695713	2505	6	93.64589	835	7.75
33	CsB-Lectin4.5	orange1.1g003391m	4	1661177..1665772	2472	6	92.31843	824	7.74
34	CsB-Lectin4.6	orange1.1g038934m	4	1684664..1687851	2256	5	84.51088	752	6.28
35	CsB-Lectin4.7	orange1.1g037333m	4	5925..6402	477	0	17.49263	159	4.96
36	CsB-Lectin4.8	orange1.1g039318m	4	1982966..1986684	2154	6	81.14631	718	7.45
37	CsB-Lectin4.9	orange1.1g005236m	4	1866644..1870056	2121	7	80.01658	707	7.73
38	CsB-Lectin4.10	orange1.1g008648m	4	1783559..1786800	1677	5	62.83662	559	7.04
39	CsB-Lectin4.11	orange1.1g046703m	4	1776739..1780553	1908	4	71.4375	636	6.24
40	CsB-Lectin4.12	orange1.1g004521m	4	1725787..1730234	2244	7	84.22815	748	6.24
41	CsB-Lectin4.13	orange1.1g004264m	4	1632319..1635887	2298	5	86.2531	766	5.66
42	CsB-Lectin4.14	orange1.1g002969m	4	1379694..1386314	2589	6	97.6331	863	6.02
43	CsB-Lectin5.1	orange1.1g003237m	5	1363515..1367504	2514	6	94.12024	838	8.6
44	CsB-Lectin5.2	orange1.1g041312m	5	1351918..1355686	2232	5	82.96567	744	5.29
45	CsB-Lectin6.1	orange1.1g042843m	6	8981..10433	1452	0	54.57688	484	8.45
46	CsB-Lectin6.2	orange1.1g036022m	6	1..814	813	0	31.04387	271	5.43
47	CsB-Lectin6.3	orange1.1g003846m	6	1583032..1586523	2376	6	89.15392	792	7.07
48	CsB-Lectin6.4	orange1.1g041702m	6	253905..257234	2523	6	94.93685	841	7.11
49	CsB-Lectin6.5	orange1.1g045315m	6	1244724..1248297	1431	3	53.85226	477	6.41
50	CsB-Lectin6.6	orange1.1g016333m	6	1281511..1285901	1176	2	44.004.03	392	5.25
51	CsB-Lectin6.7	orange1.1g036419m	6	1180370..1183611	2265	6	84.32007	755	8.3
52	CsB-Lectin6.8	orange1.1g040413m	6	1611902..1615134	2388	5	89.36793	796	6.63
53	CsB-Lectin6.9	orange1.1g003786m	6	1586575..1590199	2388	5	89.8273	796	8.08
54	CsB-Lectin6.10	orange1.1g003264m	6	1627773..1631527	2508	6	94.05896	836	8.26
55	CsB-Lectin6.11	orange1.1g003092m	6	1381101..1384495	2547	6	95.59532	849	7.16
56	CsB-Lectin6.12	orange1.1g037240m	6	1507475..1508993	921	2	34.51394	307	8.13
57	CsB-Lectin6.13	orange1.1g042187m	6	1550740..1551088	348	0	13.03983	116	9.89
58	CsB-Lectin6.14	orange1.1g014149m	6	1524447..1527865	1293	4	48.57773	431	8.09
59	CsB-Lectin6.15	orange1.1g003288m	6	1397157..1400448	2505	6	94.60412	835	6.83
60	CsB-Lectin6.16	orange1.1g003274m	6	1493897..1497542	2505	6	94.17672	835	7.89
61	CsB-Lectin6.17	orange1.1g005825m	6	1409853..1413152	2028	4	76.4913	676	8.14
62	CsB-Lectin6.18	orange1.1g041554m	6	1442787..1444092	1305	0	49.38703	435	5.67
63	CsB-Lectin6.19	orange1.1g003280m	6	1474867..1478238	2505	6	93.95059	835	6.34
64	CsB-Lectin7.1	orange1.1g013934m	7	5899..7262	1302	0	48.88107	434	5.87
65	CsB-Lectin7.2	orange1.1g036296m	7	2017..3348	1302	1	48.7893	434	5.59
66	CsB-Lectin7.3	orange1.1g043457m	7	34..1306	1272	0	48.41297	424	7.9
67	CsB-Lectin7.4	orange1.1g041743m	7	31830..33153	1323	0	49.67942	441	6.88
68	CsB-Lectin7.5	orange1.1g048464m	7	189437..190741	1272	1	47.85504	424	6.04
69	CsLysM1.1	orange1.1g036507m	1	180308..185202	1917	10	70.6922	639	6.29
70	CsLysM1.2	orange1.1g046083m	1	2031512..2036558	2373	10	87.0961	791	5.87
71	CsLysM2.1	orange1.1g005923m	2	5021752..5024254	2010	0	74.268	670	6.57
72	CsLysM2.2	orange1.1g039626m	2	2359137..2361024	1887	0	68.47634	629	4.97
73	CsLysM2.3	orange1.1g042486m	2	2353383..2355291	1908	0	69.74171	636	6.39
74	CsLysM2.4	orange1.1g037224m	2	132430..134917	1659	1	62.16937	553	8.36
75	CsLysM3.1	orange1.1g014940m	3	666786..669386	1248	4	43.19346	416	5.5
76	CsLysM3.2	orange1.1g018429m	3	193793..198011	1071	2	37.71898	357	9.28
77	CsLysM4	orange1.1g018290m	4	1134644..1138517	1077	4	38.95857	359	8.25
78	CsLysM5	orange1.1g024891m	5	381621..383556	786	1	28.8599	262	5.98
79	CsLysM6.1	orange1.1g033289m	6	500185..500620	369	0	13.25612	123	5.63
80	CsLysM6.2	orange1.1g035523m	6	1559795..1560173	378	0	13.52124	126	4.82
81	CsLysM6.3	orange1.1g044600m	6	561269..561584	315	0	11.55723	105	5.02
82	CsChitin-Bind1.1	orange1.1g035991m	1	72696..73119	423	0	15.18502	141	4.94
83	CsChitin-Bind1.2	orange1.1g020187m	1	1382269..1383964	990	2	35.192.44	330	8.27
84	CsChitin-Bind1.3	orange1.1g043941m	1	310538..310775	237	0	8.630.07	79	8.1
85	CsChitin-Bind1.4	orange1.1g023873m	1	80883..82567	831	1	29.720.17	277	4.75
86	CsLec-C	orange1.1g008216m	1	1328314..1333709	1722	4	64.82378	574	9.04
87	CsGal-B1	orange1.1g006694m	1	1243927..1247024	1908	6	71.57682	636	6.22
88	CsGal-B2	orange1.1g006860m	2	3045949..3050952	1887	6	71.09025	629	7.61
89	CsGal-B3	orange1.1g006036m	3	1349113..1352896	1992	6	75.0457	664	6.46
90	CsCalreticulin	orange1.1g009287m	1	152878..156215	1617	5	60.96163	539	4.71
91	CsJacalin1	orange1.1g007083m	1	3306879..3309850	1860	5	67.60879	620	8.98
92	CsJacalin2	orange1.1g043505m	2	824..1142	225	0	8.24503	75	5.13
93	CsJacalin3	orange1.1g020281m	3	3315760..3319295	987	2	36.26376	329	8.87
94	CsPhloem1.1	orange1.1g023485m	1	39300..41023	846	2	31.34959	282	4.98
95	CsPhloem1.2	orange1.1g036980m	1	194483..196120	591	2	23.13558	197	5.81
96	CsPhloem1.3	orange1.1g046143m	1	4186..5478	405	2	15.24674	135	8.52
97	CsPhloem1.4	orange1.1g031412m	1	233024..234243	483	2	18.01281	161	5.67
98	CsPhloem1.5	orange1.1g045697m	1	166100..166871	393	1	14.33445	131	6.87
99	CsPhloem1.6	orange1.1g039003m	1	412612..414185	861	3	32.90982	287	6.97
100	CsPhloem1.7	orange1.1g042543m	1	4111739..4112216	477	0	18.04844	159	5.59
101	CsPhloem2	orange1.1g025108m	2	1802754..1804089	774	2	29.27744	258	5.46
102	CsPhloem3.1	orange1.1g030911m	3	1808313..1809980	510	2	19.5796	170	8.95
103	CsPhloem3.2	orange1.1g040497m	3	55429..59660	888	2	33.5783	296	6.19
104	CsPhloem3.3	orange1.1g022498m	3	1638214..1640373	891	2	33.35812	297	7.57
105	CsPhloem3.4	orange1.1g022971m	3	305242..307871	870	2	32.94277	290	8.86
106	CsPhloem3.5	orange1.1g023065m	3	197287..200088	867	2	32.35918	289	8.64
107	CsGal-Lec1	orange1.1g035496m	1	496055..500425	2502	18	94.29793	834	8.97
108	CsGal-Lec2	orange1.1g045037m	2	1045866..1050451	2499	18	94.26914	833	9.31
109	CsGal-Lec3	orange1.1g041957m	3	74083..81943	2205	19	81.65729	735	5.99
110	CsGal-Lec4	orange1.1g003044m	4	273896..280140	2565	18	95.38742	855	7.1
111	CsGal-Lec5	orange1.1g003076m	5	3323553..3329348	2553	18	93.91841	851	8.33
112	CsGal-Lec6	orange1.1g002867m	6	895626..902276	2619	17	96.70592	873	8.6
113	CsGal-Lec7	orange1.1g037925m	7	1713365..1718602	2466	18	91.20659	822	6.21
114	CsGal-Lec8	orange1.1g003095m	8	1490286..1496410	2547	18	92.34346	849	7.15
115	CsGal-Lec9	orange1.1g036343m	9	1544142..1547971	2388	13	88.98474	796	7.18
116	CsGal-Lec10	orange1.1g046585m	10	1538569..1542741	2433	18	90.53306	811	6.85
117	CsGal-Lec11	orange1.1g046146m	11	178794..186659	2490	19	92.74209	830	6.31
118	CsLectinlegB1	orange1.1g007869m	1	125418..127448	1761	1	65.1513	587	8.43
119	CsLectinlegB2	orange1.1g005621m	2	1048369..1050833	2064	1	75.76026	688	5.9
120	CsLectinlegB3	orange1.1g038860m	3	155483..157524	1959	1	71.56331	653	5.82
121	CsLectinlegB4	orange1.1g035655m	4	471007..473067	2013	1	74.45276	671	6.08
122	CsLectinlegB5	orange1.1g005809m	5	467125..469529	2031	1	75.08064	677	6.22
123	CsLectinlegB6	orange1.1g048419m	6	46890..48852	1962	0	72.59838	654	6.35
124	CsLectinlegB7	orange1.1g040242m	7	563126..565112	1986	0	74.01965	662	6.35
125	CsLectinlegB8	orange1.1g044561m	8	20691..22566	1845	1	68.63275	615	7.96
126	CsLectinlegB9	orange1.1g006112m	9	3551..5597	1983	1	73.33055	661	6.48
127	CsLectinlegB10	orange1.1g045713m	10	1..823	822	0	30.53824	274	8.92
128	CsLectinlegB11	orange1.1g047725m	11	657..2438	900	1	32.79027	300	4.79
129	CsLectinlegB12	orange1.1g006668m	12	580409..582695	1911	1	70.44475	637	5.25
130	CsLectinlegB13	orange1.1g005257m	13	2227146..2229578	2118	0	78.40627	706	6.26
131	CsLectinlegB14	orange1.1g024824m	14	219005..220139	789	0	29.48157	263	9.04
132	CsLectinlegB15	orange1.1g010476m	15	209672..217385	1530	7	56.81076	510	8.63
133	CsLectinlegB16	orange1.1g008374m	16	1435696..1440010	1707	0	63.85592	569	7.72
134	CsLectinlegB17	orange1.1g006446m	17	1626586..1628545	1935	0	71.86754	645	6.31
135	CsLectinlegB18	orange1.1g047719m	18	499056..500885	1791	1	65.594.88	597	6.51
136	CsLectinlegB19	orange1.1g043702m	19	136814..138950	2136	0	76.86535	712	5.97
137	CsLectinlegB20	orange1.1g041096m	20	171050..173026	1938	1	71.71176	646	6.14
138	CsLectinlegB21	orange1.1g042831m	21	119923..121858	1935	0	72.23587	645	6.18
139	CsLectinlegB22	orange1.1g041624m	22	39464..41069	1380	2	51.36249	460	8.63
140	CsLectinlegB23	orange1.1g045189m	23	26046..26943	897	0	33.3566	299	6.66
141	CsLectinlegB24	orange1.1g040174m	24	1074..2347	999	3	37.13906	333	5.86

We identified the representative calreticulin domain in the CsCalreticulin gene family with a 1617 bp ORF. The encoded protein had a molecular weight of 60.96163 kDa, and the predicted CsCalreticulin protein exhibited higher acidic properties with a pI value of 4.71. Our analysis revealed the presence of the Jacalin conserved domain in the CsJacalin gene family. The ORF lengths ranged from 225 bp to 1860 bp, and the encoded proteins varied in length from 75 aa to 620 aa. The pI values of the CsJacalin proteins indicated that they are primarily basic, except for CsJacalin2, which exhibited the lowest pI value of 5.13, indicating acidic properties. The presence of the PP2 conserved domain confirmed the classification within the plant Phloem family. The CsPhloems exhibited ORF lengths ranging from 393bp to 891bp, corresponding to CsPhloem1.5 and CsPhloem3.3, encoding proteins of 131 and 297 aa, respectively. Among the CsPhloems, CsPhloem3.1 displayed the highest pI value of 8.95, while CsPhloem1.1 had the lowest pI value of 4.98.CsGal-Lec confirmed its identity in the plant Gal-Lec family by demonstrating Glyco_hydro_35, GHD, and Gal Lectin conserved domains. We found that CsGal-Lecs ORF ranged from 2205bp to 2619bp, belonging to the CsGal-Lec3 and CsGal-Lec6 with the coding potentiality of 735 and 873 aa, respectively. The pI values of the CsGal-Lec proteins ranged from 5.99 (acidic properties) to 9.31 (basic properties), corresponding to CsGal-Lec3 and CsGal-Lec2. LectinlegB contained the Lectin_legB and Pkinase conserved domains. The CsLectinlegBs ORF ranged from 789bp to 2136bp, with CsLectinlegB14 and CsLectinlegB19 coding for 263 and 712 aa, respectively.CsLectinlegB10 had the highest pI value of 8.92, while CsLectinlegB11 had the lowest pI value of 4.79. Previous studies have reported that snowdrop lectin (GNA) in the Amaryllidaceae family has a 333 bp ORF encoding a 157 aa protein [48], and chickpea seed lectin contains an 807 bp ORF encoding a 268 aa protein [49]. Winged bean plant lectins with varying pI values exhibited differing agglutination properties [50]. In our study, we identified 141 sweet orange genes, fewer than in Arabidopsis (199), rice (267), and soybean (309) [47]. This indicates that Cslectin gene family members are relatively smaller in sweet oranges than in other plants. However, the distribution of lectin genes can vary among species, potentially linked to their adaptation to different conditions.

### 3.2 Phylogenetic relationship of lectin proteins between sweet orange and Arabidopsis

**Fig 2:**
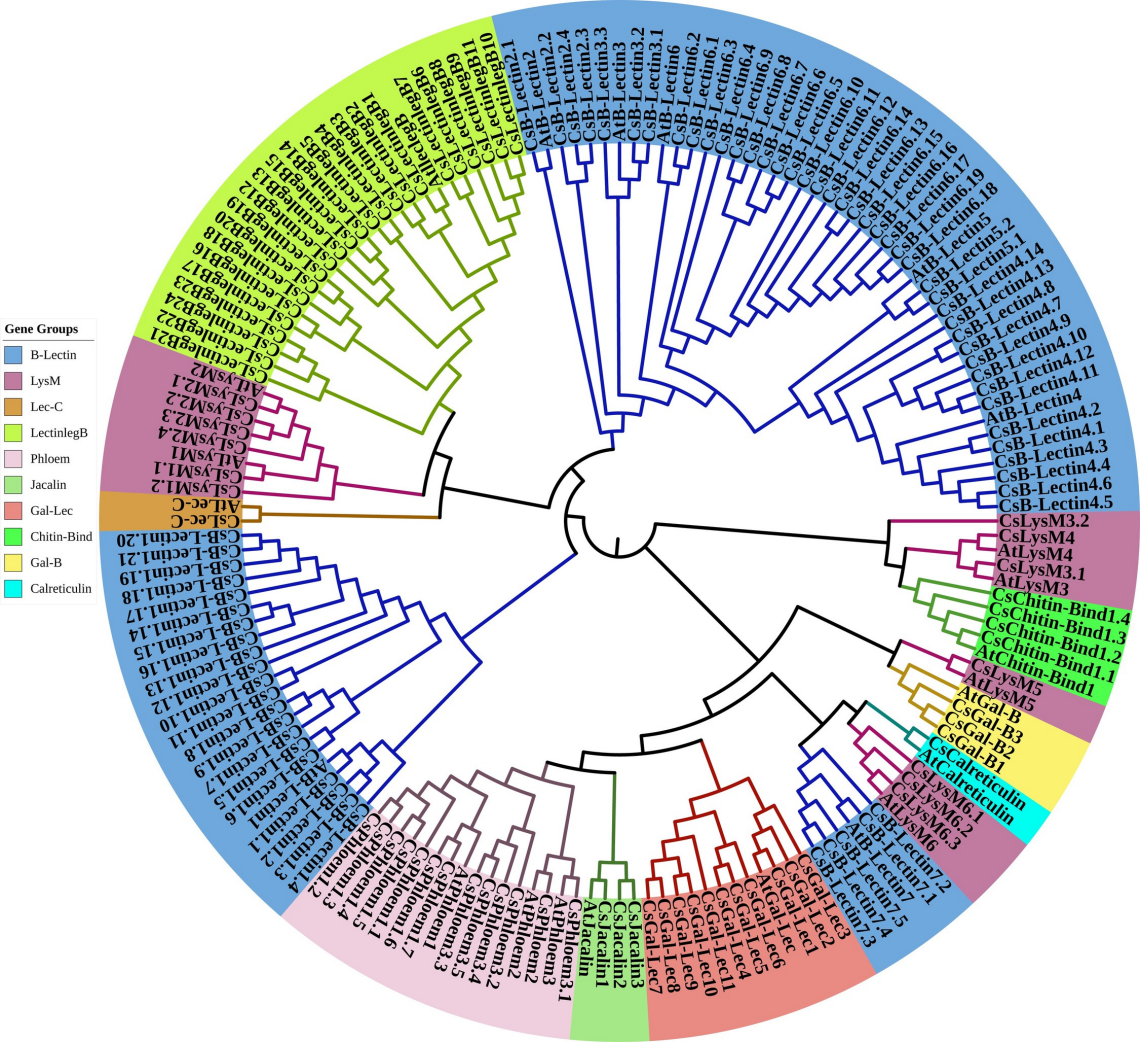
Phylogenetic tree representing the evolutionary relationship for the (I) B-Lectin proteins (II) Lysin Motif (LysM) proteins (III) Chitin-Bind-1 proteins (IV) Lec-C proteins (V) Gal-B proteins (VI) Calreticulin proteins (VII) Jacalin proteins (VIII) Phloem proteins (IX) Gal-Lec proteins and (X) LectinlegB proteins from sweet orange and Arabidopsis. The phylogenetic trees were constructed using the neighbor-joining method. Different groups present here are indicated by different colors.

The evolutionary relationships among Cslectin genes were categorized into ten clusters (B-Lectin, LysM, Chitin-Bind1, Lec-C, Gal-B, Calreticulin, Jacalin, Phloem, Gal-Lec, LectinlegB) along with Atlectin using full-length protein sequences in a phylogenetic tree analysis (Fig 2 and S4 Data). Phylogenetic analysis revealed that seven CsB-Lectin proteins (CsB-Lectin1 to CsB-Lectin7) grouped into seven distinct clusters (Group I to VII) alongside their corresponding B-Lectin proteins in Arabidopsis, supported by strong bootstrap values. These CsB-Lectin proteins (CsB-Lectin1 to CsB-Lectin7) belong to the B-Lectin1, 2, 3, 4, 5, 6, and 7 subfamilies, respectively, based on their high sequence similarity to AtB-Lectin1, 2, 3, 4, 5, 6, and 7. The B-Lectin family had the most Cslectin members, similar to rice and soybean [47].

We identified 13 LysM genes from sweet oranges that were classified into six Groups (Group I-VI). In Group I, two sweet orange proteins named CsLysM1.1 and CsLysM1.2 were clustered with AtLysM1. The two sweet orange proteins exhibited similarity with the LysM1 subfamily due to their higher sequence similarity with AtLysM1.Group II consists of four CsLysM proteins (CsLysM2.1-CsLysM2.4) along with AtLysM2 proteins. The 4 CsLysM2 proteins were clustered with the AtLysM2 subfamily due to their higher sequence similarity. Group III includes two CsLysM genes (CsLysM3.1 and CsLysM3.2) exhibiting higher sequence identity with AtLysM3. The CsLysM4 gene was clustered with AtLysM4 in Group IV, indicating its affiliation with the LysM4 subfamily due to higher sequence similarity with AtLysM4. Similarly, in Group V, CsLysM5 was clustered with AtLysM5. Moreover, the remaining three CsLysM genes (CsLysM6.1-CsLysM6.3) are classified into the LysM6 subfamily due to their higher sequence similarity with AtLysM6. LysM proteins act as pattern-recognition receptors, recognizing chitin and triggering plant immunity responses to various stresses [51]. CsLysM proteins could be induced immunity to bacterial infection based on the Arabidopsis LysM function [52]. The phylogenetic tree analysis revealed that four CsChitin-Bind-1 (CsChitin-Bind1.1- CsChitin-Bind1.4) were clustered with AtChitin-Bind1. These CsChitin-Bind1proteins are similar to the AtChitin-Bind1 subfamily based on higher sequence similarity. Previous studies have shown that Chitin-binding proteins have functional potentiality on resistance to various biotic and abiotic stresses such as drought, salinity, and cold in different crop species [53–55]. These results indicate that the CsChitin-binds proteins are predicted to be involved in signal transduction, growth, development, immune system, and responses to different stresses in the sweet orange plant. CsLec-C protein was clustered with AtLec-C and it is noted that CsLec-C is included in the Lec-C family due to its high sequence similarity with the AtLec-C gene. However, the functions of plant Lec-C remain unclear despite the involvement of the mammalian calcium-dependent lectin domain in self-/non-self-identification [56]. We found three CsGal-B proteins (CsGal-B1-CsGal-B3) which were clustered with AtGal-B. These CsGal-B proteins are similar to the Gal-B family due to their higher sequence similarity with AtGal-B. The Gal-B proteins are involved in cell growth and elongation in plants [57]. So, there is a possibility that CsGal-B proteins could be involved in responding to various growth hormone-like stimuli such as hormones.

One CsCalreticulin protein is included in the Calreticulin family due to the higher sequence similarity with the AtCalreticulin. According to previous studies, Calreticulins play an important role in plant intracellular Ca^2+^ storage, Ca^2+^ homeostasis of endoplasmic reticulum [58–60], and defense against biotrophic pathogens [61]. Calreticulins may serve as crucial molecular chaperones essential for cell survival [62–64]. Further, it may also regulate stress flexibility due to its antioxidant activity [65]. It is expected that CsCalreticulin will be involved in the growth and development of citrus plants as well as responses to biotic and abiotic stresses. Three CsJacalin proteins (CsJacalin1-CsJacalin3) were clustered together with AtJacalin proteins, indicating their membership in the Jacalin family due to their high sequence similarity with the corresponding AtJacalin proteins. Jacalin showed an advanced signal in plants during pathogen attacks [66]. The CsJacalins could be involved in the defense mechanism of sweet oranges. The phylogenetic investigations have also unveiled the presence of three distinct Phloem gene clusters, denoted as Group I, Group II, and Group III. The Phloem genes obtained from sweet orange were designated as CsPhloem1.1-CsPhloem1.7, CsPhloem2, and CsPhloem3.1-CsPhloem3.5. In Group I, 7 CsPhloem1 proteins (CsPhloem1.1-CsPhloem1.7) were clustered with the AtPhloem1 subfamily according to their higher sequence similarity with the AtPhloem1. Similarly, the CsPhloem2 protein is grouped (Group II) with the AtPhloem2 protein and exists in the Phloem2 subfamily based on the sequence similarity with AtPhloem2. Group III includes 5 CsPhloem3 genes (CsPhloem3.1-CsPhloem3.5) that showed higher sequence similarity with AtPhloem3 proteins. Phloem lectin negatively impacts the transmission of cucurbit aphid-borne yellow virus and displays antifungal activity against various fungal strains [67]. These findings indicate that CsPhloems likely play a crucial role in plant defense mechanisms. In addition, 11 CsGal-Lec proteins (CsGal-Lec1-CsGal-Lec11) clustered in the Gal-Lec family due to their sequence similarity with the AtGal-Lec. Gal-Lec family has also been found in rice, soybean, and mulberry plant species [47, 68]. We found 24 CsLectinlegBproteins (CsLectinlegB1-CsLectinlegB24), which were clustered with AtLectinlegB. These CsLectinlegBproteins are quite similar to the lectinlegB family due to their high sequence similarity with the AtLectinlegB. CsLectinlegBis is known for its antifungal, immunomodulatory, and mitogenic properties in plants [69]. For the resistance to several harmful diseases, CsLectinlegBproteins may be expected to explore and pave the way for designing and developing effective drugs.

### 3.3 Domain analysis of lectin proteins in sweet orange and Arabidopsis

**Fig 3:**
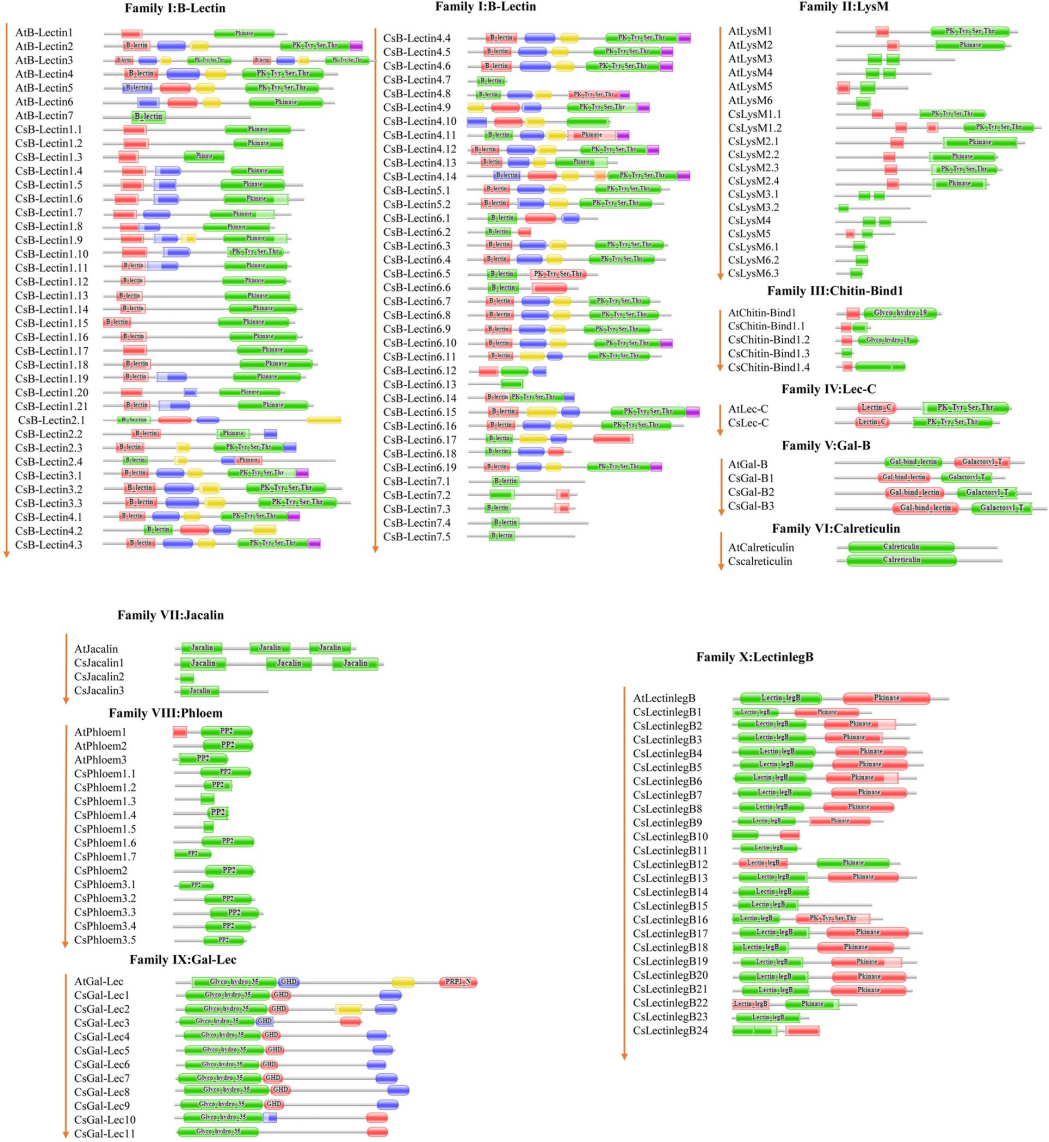
The conserved domains of the predicted Cslectin proteins were drawn by using Pfam.

Conserved domain analysis reveals a better understanding of the structure of Cslectin proteins and their classification into different protein families. Different types and numbers of typical conserved domains corresponding to the Cslectin gene family were detected by domain analysis. The domain analysis revealed strong conservation of functional domains in the B-Lectin, LysM, Chitin-Bind1, Lec-C, Gal-B, Calreticulin, Jacalin, Phloem, Gal-Lec, and LectinlegB families in both sweet orange and Arabidopsis (Fig 3). Apart from the carbohydrate-binding domain, the conserved domains in the lectin gene family exhibit diverse biological activities in plants [70]. All significant conserved domains: Pkinase, B_lectin, Pk_Tyr_Ser_Thr, S locus glycop, PAN2, and DUF3403 were exhibited in the CsB-Lectin proteins. The S_Locus_Glyco_protein domain associated with the B-Lectin family was involved in arbitrating the self-incompatibility responses in *Brassicasp* [71].

The presence of the Kinase domain along with the PAN2 domain in the B-Lectin family suggests a potential role in mediating interactions between proteins and sugars [68]. *OsSIK2* carrying B_lectin, S-_Locus_Glycoprotein, PAN domain, and Ser/Thr kinase domain which are involved in delaying leaf senescence and enhancing drought and salt tolerance [72]. Most of the proteins with LysM motifs act as pattern-recognition receptors by recognizing the chitin which increases plant immunity [51]. LysM receptor-like kinases also regulate the symbiosis of *rhizobium*-legume as well as arbuscular mycorrhizae [73]. In plants, LysM domains of LysM proteins remained highly diversified, and six distinct types of LysM existed in sweet orange. LysM, Pkinase, and PK_Tyr_Ser_Thr domains were observed in CsLysM and AtLysM. Both sweet orange and Arabidopsis have a single member in the Lec-C family, each of which contains both Pk_Tyr_Ser_Thr and Lectin_C domains. Plant Lectin_C domains share 30% sequence identity with animal domains, suggesting a potential role as C-type lectins [47]. Proteins carrying C-type lectin domains were involved in inducing immunologic response against pathogens and apoptosis [74, 75]. Members of the LectinlegB family in sweet orange and Arabidopsis included the Lectin_legB and Pkinase domains. PK_Tyr_Ser_Thr domain was exhibited in a single member of sweet orange named CslectinlegB16. In our analysis, the typical calreticulin domain was predicted in sweet orange Calreticulin proteins that demonstrated the similarity with Arabidopsis Calreticulin domain. In the endoplasmic reticulum, part of the quality control system for glycoproteins is formed by Calreticulin [76, 77]. Calreticulin proteins were involved in Ca^2+^ signaling and protein folding. These proteins also involved in plant growth and development as well as the responses to biotic and abiotic stresses [78]. Sweet orange Chitin-Bind1 family members contain Glyco hydro 19 and Chitin-Bind1 domains. In wheat embryos, a chitin-binding lectin called WGA plays a role in defending seedlings during fungal attacks [79]. Probably, the CsChitin-Bind1 protein can perform a crucial role associated with defensive strategy in sweet orange. Further characterization of this protein will be needed to clarify the fact. The identified phloem domain in sweet orange showed a similarity with the corresponding Arabidopsis phloem domain. The phloem domain (Phloem protein 2) found in angiosperms is linked to the F-box domain and is thought to be involved in protein degradation via sugar-protein interactions [80]. The predicted Jacalin functional domains of sweet orange also showed similarity with the respective Jacalin of Arabidopsis. CsJacalin1 had three Jacalin domains, while the other two (CsJacalin2 and CsJacalin3) had only one. Jacalin-related lectins played a significant role in the growth and defense and stress responses of plants [25, 81]. Jacalin domain in Ta-JA1, jacalin-like lectin in wheat exhibited agglutinating activity and resistance to a pathogen [82]. *O*-Glycosyl hydrolase domain, like Glyco_hydro_35 involved in the hydrolysis of the glycosidic bonds, were identified in all the members of the CsGal-Lec. Previous studies have shown that the presence of Glyco_hydro_35 domains was present in 69.2% of the total members of Arabidopsis and every single member of the soybean and rice Gal-Lec family [47]. We predicted another *β*-galactosidase domain, betaGal_dom4_5 in only CsGal-Lec 2. CsGal-Lec also demonstrated GHD and Gal Lectin domains. Galactosyl_T domain was reported in each of the members of Gal-binding lectin family in soybean, rice and Arabidopsis [47]. This conserved domain was found in all three out of three members (CsGal-B1-CsGal-B3) of the respective family in sweet orange.This finding suggests that typical conserved domain of each Cslectin family members may have important biological functions with desired agronomic traits which could be analyzed in detail in wet-lab conditions to improve the sweet orange cultivar in the future.

### 3.4 Motif analysis of lectin proteins in sweet orange and Arabidopsis

**Fig 4:**
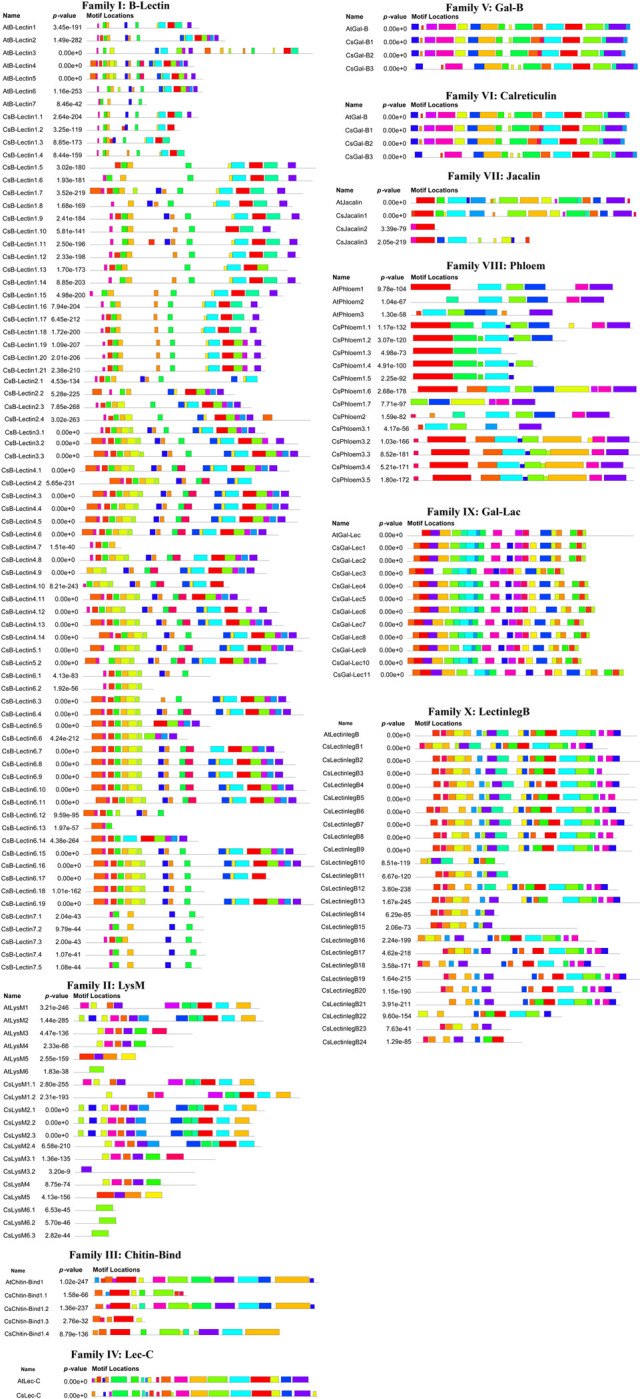
The conserved motifs of the predicted Cslectin protein families are drawn by MEME-suite (a maximum of 19 motifs are displayed). Different colors indicated different motifs allocated in the domains of the predicted proteins.

Motif distributions may vary among the protein sequences of target genes which serve as a key reporter to observe the functional diversity of various subfamilies [38]. In our analysis, a maximum of 19 motifs in both proteins of CsB-Lectin and AtB-Lectin were predicted (Fig 4). There is a possibility that CsB-Lectin7 will appear highly functional like AtB-Lectin7. However, CsB-Lectin7 comprises 5 motifs that are very similar to the paralogs AtB-Lectin7. Besides this, 18 motifs were found in two members of the CsB-Lectin5 subfamily, whereas AtB-Lectin5 contained 17 motifs.

CsB-Lectin2 members exhibited fewer motifs (7–14 motifs) compared to the 15 motifs found in AtB-Lectin2.The motif number is close to similar (approximately 17–18 motifs) in CsB-Lectin3 and AtB-Lectin3 (16 motifs). Most of the members of CsB-Lectin1, CsB-Lectin4, and CsB-Lectin6 will probably be functionally active like AtB-Lectin1 (10 motifs), AtB-Lectin4 (19 motifs) and AtB-Lectin6 (15 motifs) proteins, respectively. The presence of varying motif counts among members suggests potential functional diversity between sweet orange and Arabidopsis within the CsB-Lectin family. We also observed that CsLysM proteins had a maximum of 13 motifs. Among them, CsLysM1 (CsLysM1.1), CsLysM2 (CsLysM2.1-CsLysM2.3), CsLysM3 (CsLysM3.1), CsLysM4, CsLysM5, and CsLysM6 (CsLysM6.1-CsLysM6.3) exhibited higher conservation with their respective paralogs in AtLysM1 (10 motifs), AtLysM2 (13 motifs), AtLysM3 (6 motifs), AtLysM4 (5 motifs), AtLysM5 (4 motifs), and AtLysM6 (1 motif) proteins. The findings suggest that the CsLysM4, CsLysM5, and CsLysM6 proteins, due to their high homology, are likely to exhibit functional similarities similar to the corresponding motifs. However, the variability of motifs was observed in CsLysM1.1, CsLysM2.4, and CsLysM3.2. This motif versatility in CsLysM and AtLysM will be responsible for the variable function. We predicted 5–14 numbers of conserved motifs in the CsChitin-Bind1 family compared to AtChitin-Bind1 (15 motifs). CsLec-C contained all the 20 motifs found in paralog AtLec-C. Based on our findings, CsGal-B proteins (CsGal-B1, CsGal-B2, and CsGal-B3 contained 18, 17, and 16 motifs, respectively) showed discrepancy with the paralog AtGal-B (17 motifs).

CsCalreticulin exhibited higher conservation (20 motifs) with the paralog AtCalreticulin (20 motifs) and were expected to exhibit functional similarities. CsJacalin proteins (CsJacalin1, CsJacalin2, and CsJacalin3 contained 17, 2, and 9 motifs, respectively) showed discrepancy with the paralog AtJacalin (17 motifs). Members of CsPhloem1 (3–9 motifs), CsPhloem2 (8 motifs), CsPhloem3 (5–10 motifs) showed diversity in the number of motifs as compared with AtPhloem1 (6 motifs), AtPhloem2 (6 motifs) and AtPhloem3 (7motifs), respectively. The variation in motif distribution indicates diverse functional roles. We found that the motif number in CsGal-Lec members is higher than in AtGal-Lec (17 motifs) proteins. We also detected 3 motifs in most of the members of CsGal-Lec that were absent in AtGal-Lec. Additionally, we identified up to 20 motifs in the CsLectinlegB7 protein, which are analogous to its paralog AtLectinlegB. It is conceivable that CsLectinlegB7 shares functional similarities with AtLectinlegB due to their identical motif count. However, among the other 23 CsLectinlegB members (CsLectinlegB1-CsLectinlegB6, and CsLectinlegB8-CsLectinlegB24), many motifs present in AtLectinlegB were often absent, suggesting potential variations in their functional roles. The motif analysis indicates that particular motifs might be accountable for delineating the distinct functional roles of genes within various subfamilies. Notably, the absence of certain motifs in select genes may contribute to functional divergence. Moreover, Cslectin genes within the same groups or subgroups displayed analogous patterns of motif distribution, highlighting their distinct yet closely related evolutionary relationships among individual genes.

### 3.5 Analysis of gene structure of lectin proteins in sweet orange and Arabidopsis

**Fig 5:**
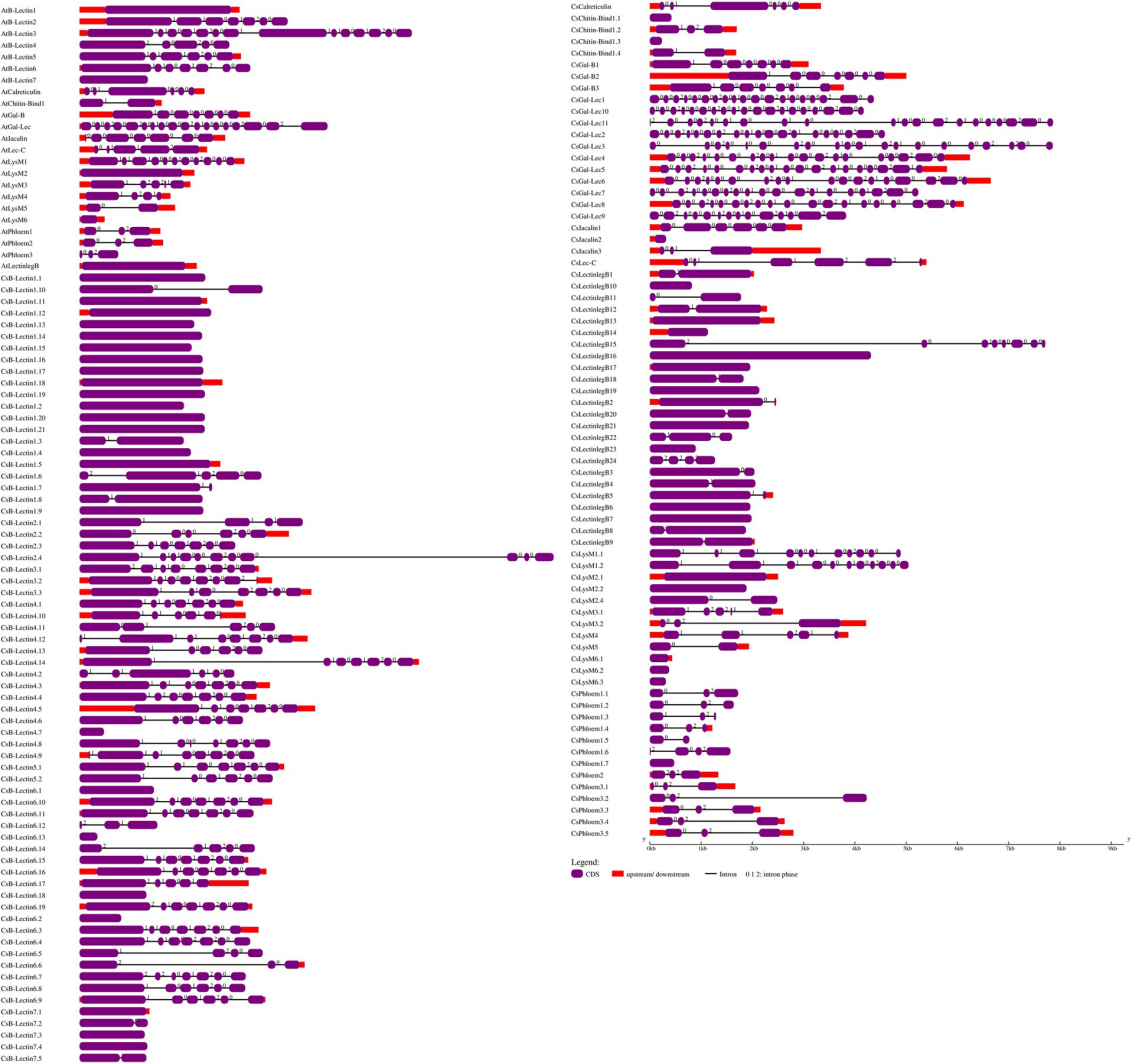
Gene structure of the predicted Cslectin genes in sweet orange with Arabidopsis by using Gene Structure Display Server (GSDS 2.0,http://gsds.gao-lab.org/).

Gene structure (exon-intron) means the arrangement of particular sequence components within a gene, which reflects the key indicator of the evolutionary relationship of the targeted gene family among the organisms or genes [83, 84]. Mostly, gene carries the information needed for survival and reproduction [85, 86]. The identified all Cslectin genes exhibited well-conserved gene structure and structural similarity with the Arabidopsis corresponding lectin genes based on gene structure analysis (Fig 5). In our analysis, the greatest number of Cslectins were found to carry introns. In the CsB-Lectin family, 24 members out of 68 members were identified to be intronless and the majority of those members belonged to the B-Lectin1 subfamily. CsB-Lectin3.2, CsB-Lectin4.9, and CsB-Lectin4.12 were identified to contain the maximum intron numbers (7) among all the members of the CsB-Lectin family. The structure of CsB-Lectin genes resembled that of the AtB-Lectin genes, although it is important to note that all members of CsB-Lectin3 exhibited fewer introns compared to AtB-Lectin3. The CsLysMs exhibited maximum variable numbers of intron (1–10) which were nearly identical to the gene structures of AtLysMs. Close similarity was also detected between CsChitin-Bind1 and AtChitin-Bind1 even though CsChitin-Bind1.1 and CsChitin-Bind1.3 remained intron less. The three CsGal-B members shared a structural similarity with AtGal-B, as they all featured 7 exons and 6 introns (Fig 5 and Table 2).

We also found CsCalreticulin having an identical number of 6 exons and 5 introns with AtCalreticulin. Out of three members comprising the Jacalin family, two members contained 5, and 2 introns, respectively which showed close similarity with the AtJacalin except for CsJacalin2 which was found to be intronless. Out of 13 CsPhloem genes, 10 CsPhloem genes exhibited 2 introns while CsPhloem1.5 and CsPhloem1.6 carried 1 and 3 introns, respectively and CsPhloem1.7 was intronless. CsPhloems intron numbers revealed similarity with AtPhloems. Likewise, eleven CsGal-Lec genes displayed 13–19 numbers of the intron in the gene structure that had demonstrated similarity with the AtGal-Lec gene structure. Among all the predicted Cslectin genes, CsGal-Lec11 from this family contains the largest number of introns (19). Gene structure similarity was also observed between LectinlegB genes of Arabidopsis and sweet orange. Significant number of members of LectinlegB (LectinlegB6, LectinlegB7, LectinlegB10, LectinlegB13, LectinlegB14, LectinlegB16, LectinlegB17, LectinlegB19, LectinlegB21 and LectinlegB23) were intron less genes, while CsLectinlegB15 from this family contained maximum 8 exons and 7 introns. In the term of exon-intron organization, substantial heterogeneity was detected in all different lectin gene families and quite similar functional roles were suggested due to the similar gene structure of identified CsB-Lectin, CsLysM, CsChitin-Bind-1, CsLec-C, CsGal-B, CsCalreticulin, CsJacalin, CsPhloem, CsGal-Lec, and CsLectinlegB with their orthologs Arabidopsis.

### 3.6 Analysis of chromosomal location of lectin genes in sweet orange

**Fig 6:**
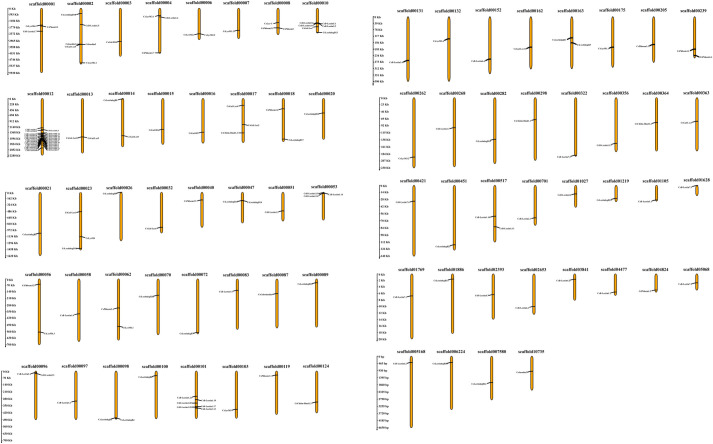
The chromosomal location of the predicted Cslectin genes. The chromosomal length indicating scale is provided on the left. The ChrUn means the unknown chromosome.

Chromosomal position analysis provides precise information about the location of genes on a chromosome. This occurrence guarantees the ability to determine the separation between one gene and others, whether they are on the same chromosome or different ones. The location of an independent gene on a chromosome can signal gene duplication, a significant factor in ancestral evolution and gene variation [87]. In this study, we created a chromosomal map to pinpoint the exact positions of the identified Cslecting gene family members. The chromosomal analysis showed 10 Cslectin gene family members unevenly distributed across 76 scaffolds in the sweet orange genome (Fig 6). The sixty-eight CsB-Lectin genes were located in 30 scaffolds. Thirteen CsLysM genes were distributed in 12 scaffolds. CsChitin-Bind1 genes were positioned in only four scaffolds. We also found CsLec-C and CsCalreticulin genes in only one scaffold, respectively. CsGal-B appeared in three scaffolds. Again, we predicted the CsJacalins in two scaffolds. CsPhloems were distributed in 11 independent scaffolds. Moreover, we observed CsGal-Lec genes in nine scaffolds. Lastly, the identified 24 CsLectinlegB were detected throughout the 21 scaffolds. According to Mendelian inheritance principles, this distribution raises the likelihood of independent segregation during cell division [88]. Recombination events among Cslectin genes are likely to take place. The sequence similarities observed among different Cslectin genes imply that the Cslectin gene family in sweet oranges may have undergone duplication or recombination events in the course of evolution. Gene and chromosomal duplications play a vital role in the differentiation of gene families in plant evolution [87].We also noted that 23 gene pairs were situated close to each other within their respective genomic positions on scaffolds 00002, 00006, 00008, 00010, 00012, 00013, 00047, 00053, 00096, 00098, and 00101. The proximity of the identified genes within the genome implies the existence of multiple expression patterns for the gene pairs. This observation indicates that various Cslectin genes located on the same chromosome may code for proteins with diverse functions.

### 3.7 Analysis of gene ontology of lectin genes in sweet orange

**Fig 7:**
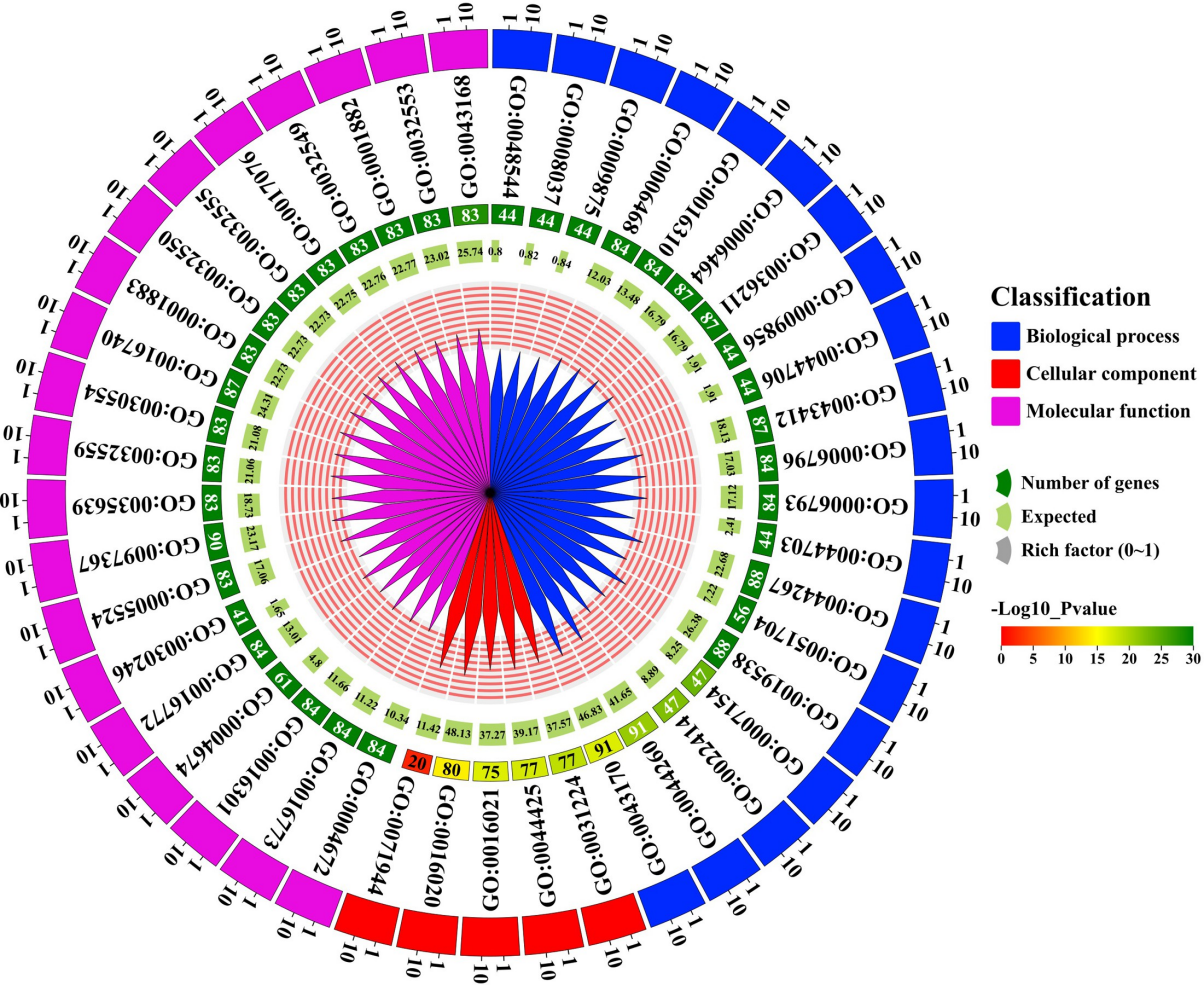
The circular heatmap for the predicted GO terms corresponding to the predicted Cslectin genes is presented for biological process, cellular components, and molecular function, whether the genes are associated or not. The *p*-value matching the GO terms is shown in the heatmap, using log10 (*p*-value).

To explore the biological roles of the predicted Cslectin genes, we carried out a detailed gene ontology (GO) enrichment analysis. The Gene ontology (GO) study assembles information from PlantTFDB 4.0 databases. The GO analysis forecasts either the position or the operative similarities of the expressed genes within the cells [89, 90]. The GO analysis results showed that the predicted GO ID of the identified Cslectin genes was categorized into three: biological processes, cellular components, and molecular functions (Fig 7 and S5 Data). A total of 20 GO IDs for Cslectin genes were selected based on their p-values, encompassing 44 genes involved in various biological processes. The GO analysis results indicated that 44 genes play a role in recognizing pollen (GO:0048544, *p*-value: 1.00E-30), cell recognition (GO:0008037, *p*-value: 1.00E-30), pollen-pistil interaction, pollination, and multicellular organism processes (GO:0044706, *p*-value: 1.00E-30). Additionally, 84 genes were linked to protein phosphorylation (GO:0006468, *p*-value: 1.00E-30), and multicellular organism processes, and 87 genes were associated with macromolecule modification (GO:0043412, *p*-value: 1.00E-30) as well as multi-organism reproductive processes. We identified 5 Cslectin GO IDs which are involved in various cellular components. As an example, we found that 77, 77, 75, 80, and 20 genes were associated with the following cellular components based on their respective p-values: intrinsic component of membrane (GO:0031224, *p*-value: 1.80E-19), membrane part (GO:0044425, *p*-value: 3.60E-18), integral component of membrane (GO:0016021, *p*-value:1.20E-17), membrane (GO:0016020, *p*-value: 7.90E-15), and cell periphery (GO:0071944, *p*-value: 0.0078). We also identified 20 Cslectin GO IDs based on their associated p-values, revealing their molecular functions. A total of 84 genes were observed to be associated with protein kinases (GO:0004672, *p*-value: 1E-30), phosphotransferase activity (GO:0016773, *p*-value: 1E-30), kinase activity (GO:0016301, *p*-value: 1E-30). Moreover, we also observed 61 genes to be responsible for protein serine/threonine kinase activity (GO:0004674, *p*-value: 1E-30), while 84 genes were associated withtransferase activity (GO:0016772, *p*-value: 1E-30).

Additionally, many Cslectin genes were assumed to play significant roles in response to external biotic stimulus, response to other organisms, response to biotic stimulus, and response to external stimulus. Previous studies suggested the involvement of lectin genes in responses to external biotic and abiotic stimuli [53–55, 61, 67]. GO analysis disclosed the involvement of predicted genes in different biological functions. Phytoalexins were observed to perform a key function in interactions with enzymes and storage proteins [91]. The plant LysM proteins play a vital role in recognizing chitin which triggers the immune response in plants [51]. The involvement of genes (allocated for cellular processes GO terms in the cytoplasm, membrane-bound organelles, or endoplasmic reticulum) supported the existing studies proposing the localization of lectin in nuclear and cytoplasmic compartment apart from the secretory pathway [90]. The findings corresponded well with our data on the distribution of functional domains in sweet orange lectins. The pointed-out GOs in this study would open the way for detecting new roles of these lectin genes.

### 3.8 Sub-cellular localization of lectin proteins in sweet orange

**Fig 8:**
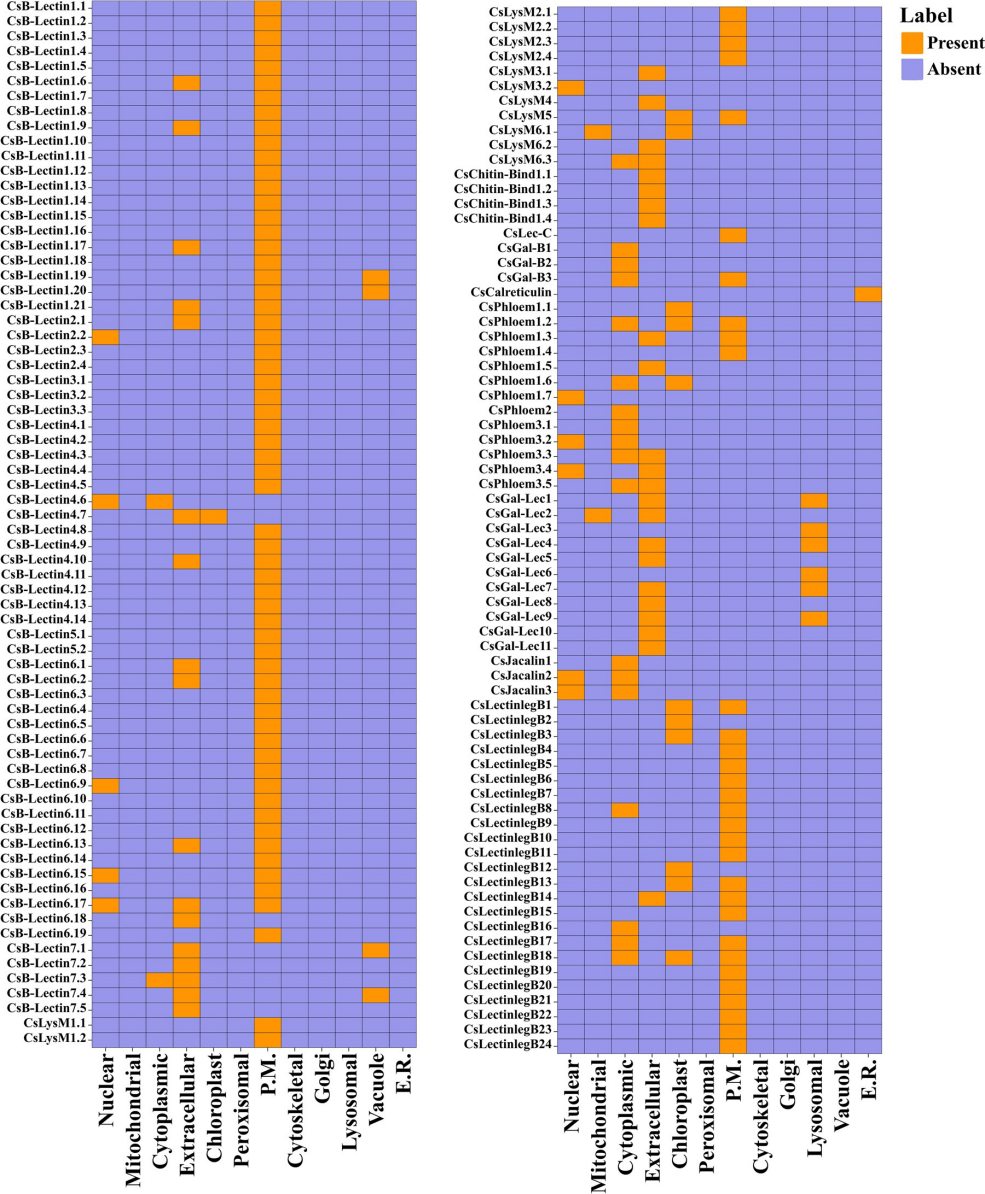
Sub-cellular localization analysis for the CsB-Lectin, CsLysM, CsChitin-Bind-1, CsLec-C, CsGal-B, CsCalreticulin, CsJacalin, CsPhloem, CsGal-Lec and CsLectinlegB proteins. In this study, reported proteins were analyzed in nuclear, mitochondrial, cytoplasmic, extracellular, chloroplast, peroxisomal, plasma-membrane (P.M.), cytoskeletal, golgi, lysosomal, vacuole, and endoplasmic reticulum (E.R.) regions.

The sub-cellular localization of particular proteins has a direct relation with the biological functions in eukaryotic cells. The annotation of sub-cellular localization may provide us the information about their functional roles at the cellular level [92, 93]. In our analysis, we predicted the presence of lectin protein signals in various significant cell organelles within sweet orange, including the nucleus, mitochondria, cytoplasm, extracellular space, chloroplast, plasma membrane, lysosome, vacuole, and endoplasmic reticulum (ER). Interestingly, the majority of these proteins were found to be expressed in the plasma membrane, as shown in Fig 8. Furthermore, CsB-Lectin proteins were predicted to be distributed across multiple cellular compartments, including the nucleus, cytoplasm, chloroplast, plasma membrane, vacuole, and extracellular region. A previous study has shown the location of classical lectin in the vacuole and apoplast, whereas the inducible lectins are found in the cytosol and nucleus [93]. We found that CsLysM proteins were localized in nuclear, mitochondrial, cytoplasmic, extracellular, chloroplast, and plasma-membrane areas. Remarkably, all CsChitin-Bind1 proteins appeared in the extracellular zone. CsLec-C and CsCalreticulin were observed to occur respectively in plasma-membrane and ER. There was a prediction that CsGal-B proteins were present mostly in cytoplasm and sometimes in plasma-membrane. CsJacalin occurs in the nucleus and cytoplasm. Sub-cellular localization of the EUL lectin gene in rice (showed response to drought and salt stress, ABA treatment, and pathogen infection) was located in the nucleus and cytoplasm as well [94]. CsPhloem proteins were localized in the nucleus, cytoplasm, extracellular, chloroplast, and plasma-membrane. Plasma membrane proteins perform several functions such as carrying nutrients, receiving and translating chemical signals, and anchoring cells in a specific location [95]. CsGal-Lec proteins were likely to appear in the extracellular and lysosomal regions. Only CsGal-Lec2 appeared in mitochondria. The gene present in mitochondria may act as a signal mediator and thus may participate not only in the development but also in the stress response of plants [96]. CsLectinlegBproteins were found to be abundant in the plasma-membrane. Except this, it was also found in chloroplast, extracellular, and cytoplasmic regions. Notably, most of the metabolism activity in plants takes place in the cytosol [97]. The predicted proteins in sweet orange located in the cytoplasmic region may be conducting the transformation of energy needed for germination, healing, and reproduction. This analysis indicates that Cslectin protein signals are localized in the specific organ and a greater number of Cslectin genes may show signals in the intracellular organ, while other genes may be extracellular and could be involved in various important functions related to microenvironments.

### 3.9 Regulatory relationship between transcription factors and lectin genes in sweet orange

**Fig 9:**
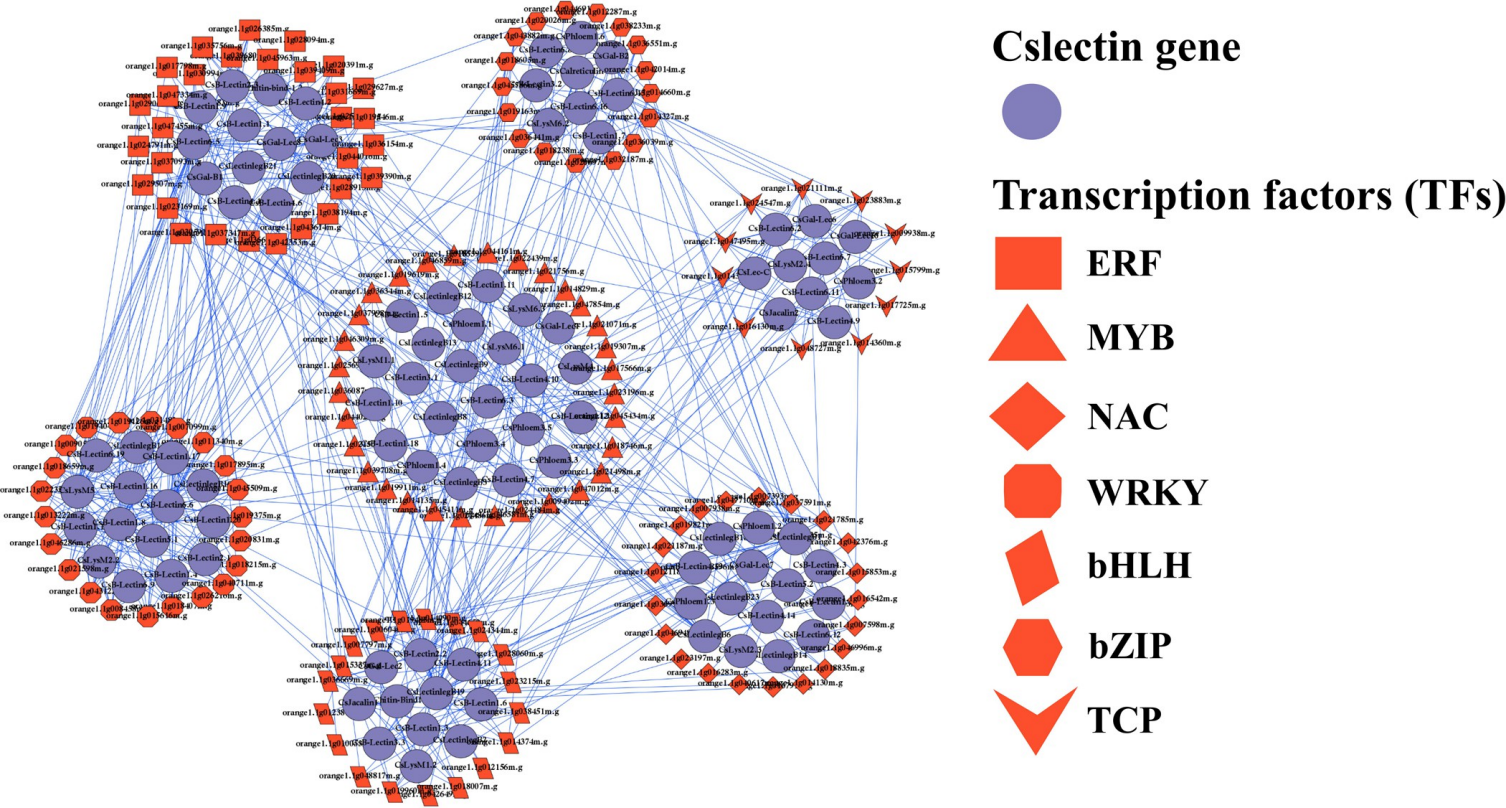
The regulatory network among the TFs and the predicted CsB-Lectin, CsLysM, CsChitin-Bind1, CsLec-C, CsGal-B, CsCalreticulin, CsJacalin, CsPhloem, CsGal-Lec and CsLectinlegB genes.

**Fig 10:**
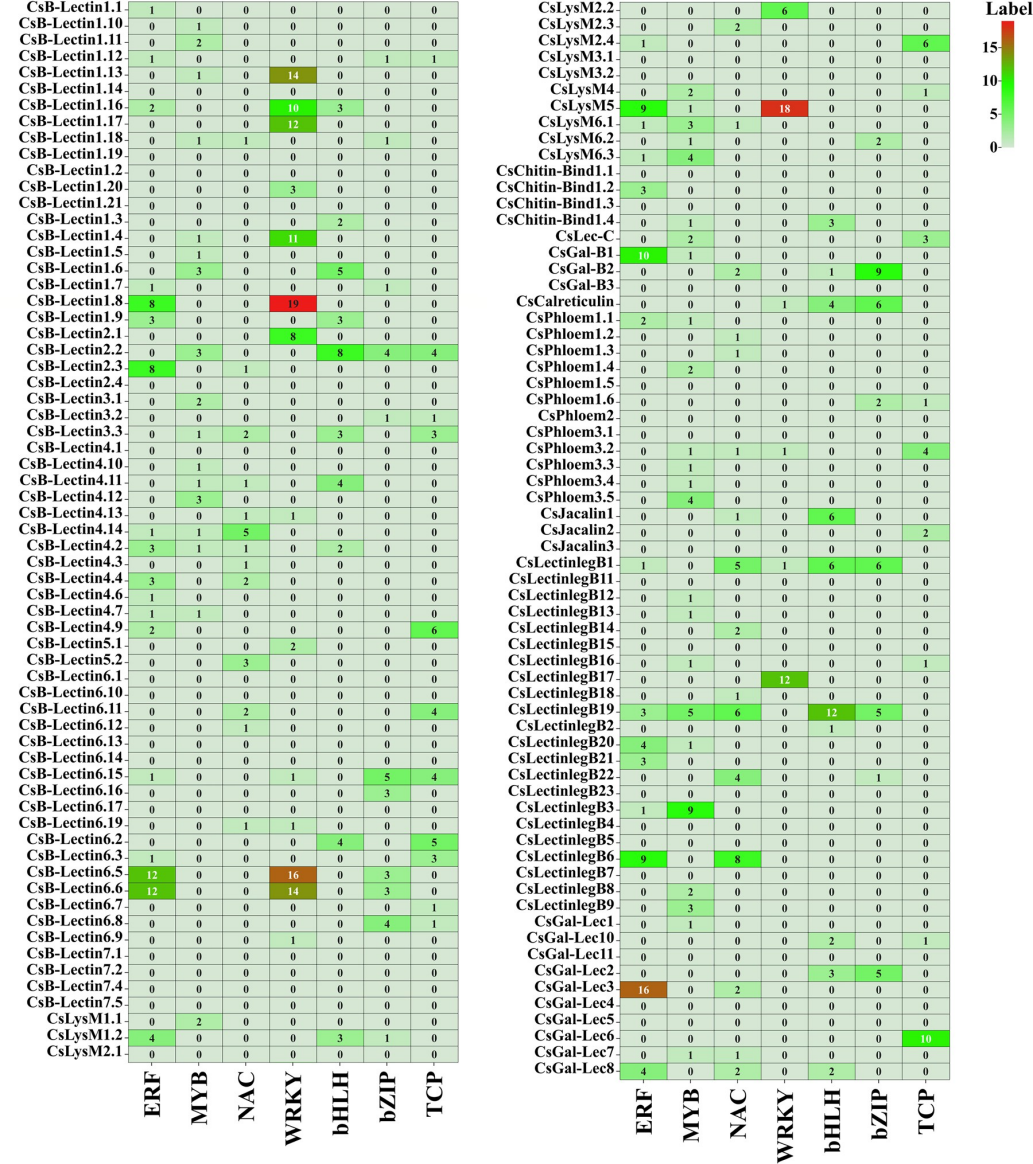
Cslectin gene-mediated sub-network for ERF, MYB, NAC, WRKY, bHLH, bZIP, and TCP TFs families which are expressed as heatmap. The related number of TFs with the reported Cslectin genes are represented inside the square boxes of a heatmap.

In plants, transcription factors (TFs) are associated with numerous important biological functions such as growth, metabolism, development, defense against microbial infection, and responses to different stresses [98–100]. Many diverse TFs, e.g., ERF, Dof, MYB, AP2/EREBP, BOS1, MIKC_MADS, NAC, and WRKY were present in plants that acted as a molecular switch of some particular genes under different developmental conditions and stresses [99, 101–104]. In this study, in total 278 unique TFs were identified that could regulate the candidate lectin genes identified in the sweet orange genome (Figs 9 and 10 and S6 Data). We divided the identified TFs into 36 groups based on TFs families. The top-ranked seven TF families; ERF, MYB, NAC, WRKY, bHLH, bZIP, and TCP families included 31 (11.15%), 31 (11.15%), 24 (8.63%), 22 (7.91%), 19 (6.83%), 17 (6.11%), and 11 (3.95%) TFs respectively, which included approximately 59.35% of the total identified TFs. According to the network analysis, the recognized TF family exhibited a certain structure and connected to the candidate lectin genes. In our analysis, we established the regulatory connections of the ERF family with seventeen CsB-Lectin genes, five CsLysM genes, one CsGal-B gene, two CsGal-Lec genes, one CsPhloem gene, and six CsLectinlegB genes. The ERF predominantly exhibited an association with CsGal-Lec3. The MYB transcription factor family was connected to sixteen CsB-Lectin genes, four CsLysM genes, one CsChitin-Bind1 gene, one CsGal-B gene, one CsLec-C gene, four CsPhloem genes, two CsGal-Lec genes, and eight CsLectinlegB genes. Moreover, we also detected the relationship between MYB and CsLectinlegB3, and NAC was associated with CsLectinlegB6. The NAC TF was associated with thirteen CsB-Lectin genes, two CsLysM genes, one CsGal-B gene, three CsGal-Lec genes, one CsJacalin gene, three CsPhloem gene, and six CsLectinlegB genes. The TCP TF built the regulatory relationship with eleven CsB-Lectin genes, two CsLysM genes, one CsLec-C gene, one CsJacalin gene, two CsPhloem genes, two CsGal-Lec genes, and one CsLectinlegB genes. The WRKY was dominantly associated with the lectin gene CsB-Lectin1.8. Additionally, WRKY showed associations with seven CsB-Lectin genes, one CsLysM gene, and one CsLectinlegB gene. We also identified a regulatory connection between the WRKY transcription factor family and fourteen CsB-Lectin genes, two CsLysM genes, the CsCalreticulin gene, one CsPhloem gene, and two CsLectinlegB genes. Additionally, bHLH was linked to CsB-Lectin2.2 and CsLectinlegB19. Our analysis also revealed that the TF familybHLH was connected with nine CsB-Lectin genes, one CsLysM gene, one CsChitin-Bind-1 gene, one CsGal-B gene, three CsGal-Lec gene, one CsCalreticulin gene, one CsJacalin genes, and three CsLectinlegB genes. The bZIP TF was linked to ten CsB-Lectin genes, two CsLysM genes, one CsGal-B gene, one CsGal-Lec gene, one CsCalreticulin gene, three CsLectinlegB gene, and one CsPhloem genes. The TCP TF built the regulatory relationship with eleven CsB-Lectin genes, two CsLysM genes, one CsLec-C gene, one CsJacalin gene, two CsPhloem genes, two CsGal-Lec genes, and one CslectinlegB genes.

Members of the NAC family play various roles in plant life cycles, including regulating salt-responsive flowering through Flowering Locus T (FT) in Arabidopsis [105], negatively controlling xylem fiber formation [106], and inducing lateral root development [107]. *CsNAC1*, found in *Citrus sp*., is expressed in leaves and shoot meristem tissue and is associated with different abiotic stress factors such as ABA, salt stress, and cold [108]. ERF family members are linked to hormonal signal transduction, metabolic regulation, and responses to both biotic and abiotic factors in various plant species [109–113]. Fruit development in sweet oranges requires the involvement of many different TF families including WRKY, MYB, and bHLH [114, 115]. In sweet orange, MYB transcription factors *CsMYB85*, *CsMYB330*, and *CsMYB308* regulate lignin biosynthesis [116, 117]. *CsBZIP40* positively functions in citrus bacterial canker (CBC) response which is caused by *Xanthomonas citri* subsp. citri (Xcc) [118]. TCP TFs family is associated with the regulation of features in different plant species development that includes branching and flower symmetry [119]. *CsTCP3*, *CsTCP9*, and *CsTCP13* are involved in the development of leaves; *CsTCP12* and *CsTCP14* play significant roles in the development of leaves and thorns and branching of shoots, and *CsTCP15* take part in the development of leaves, thorns, or stem [120]. Transcription factors bHLH carrying a conserved bHLH domain play a significant role in plant biological processes [121, 122]. Proteins encoded by the WRKY gene family are involved in various physiological and developmental processes, as well as in plant defense mechanisms during pathogen attacks [123]. Through the expression of *CsLOB*1 and the promotion of cell expansion, the *CsWRKY22* transcription factor regulates susceptibility to canker [124]. These results suggest that the enrichment of diverse and distinct TF families can be a potential source of significant functional variability among the candidate Cslectin genes.

### 3.10 Analysis of cis-acting regulatory elements (CAREs) of lectin genes in sweet orange

**Fig 11:**
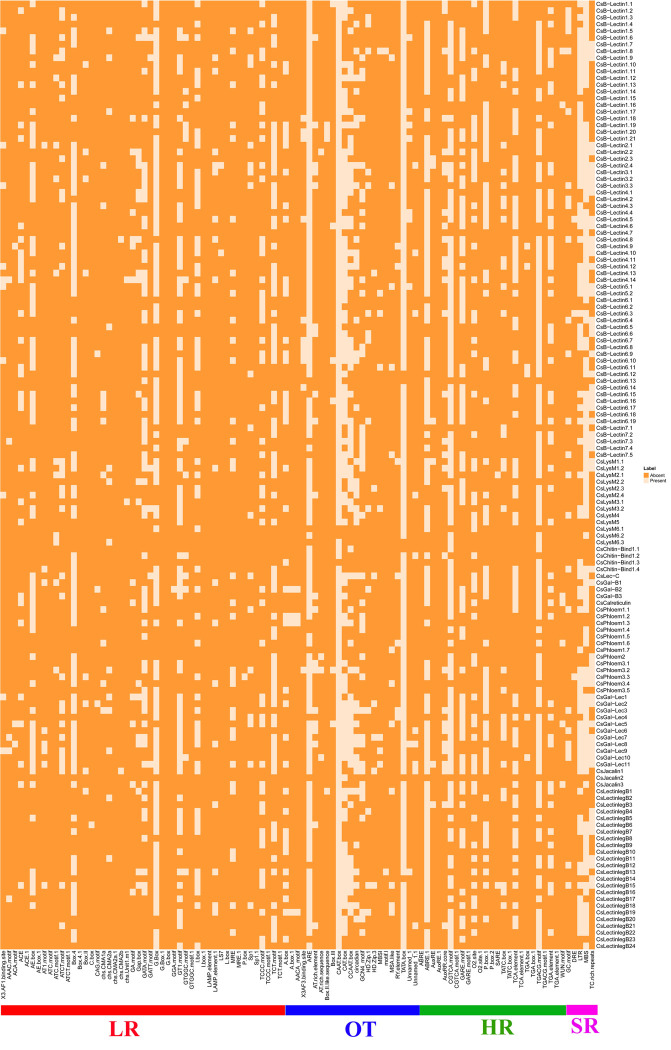
The CAREs in the upstream promoter region of reported Cslectin genes, respectively. The deep color represents the presence of that element with the corresponding genes.

The cis-acting regulatory elements (CAREs) are usually 5–20 bp non-coding DNA motifs. TFs and other regulatory molecules may trigger the process of transcription and control gene regulation by binding the target sites of CAREs [125]. In plants, the cis-elements are involved in the development and defense against various types of biotic and abiotic stresses [125, 126]. The purpose of the conduction of CARE analysis was to look for the functional diversity of the motifs located in the promoter region of the predicted lectin genes in sweet orange. The associated CAREs in sweet orange were categorized into four groups: light-responsive (LR), stress-responsive (SR), hormone-responsive (HR), and others (OT), as per the PlantCARE database (Fig 11 and S7 Data).

The CAREs analysis result revealed that the maximum number of cis-regulatory motifs exist in the LR group. Among the LR motif, 3AF1 binding site, AAAC motif, ACA motif, AT1 motif, ATC-motif, box-II, C box, CAG motif, chs CMA2b, chs unit1m1, GA motif, GATT-motif, G box 1, GGA motif, GTGGC motif, LAMP element, LS7 and L-box were mainly shared by the vast numbers of predicted lectin genes in sweet orange. Except this, some additional significant LR motifs were also found to be shared that includingACE, AE box, ATCT motif, box-4, chs CMA1a, chs CMA2a, Gap box, GATA motif, G-box, GT1 motif, I box, MRE, Sp1, TCCC motif and TCT motif. Photosynthesis, which is a crucial physiological parameter is related to light response that usually occurs in plant leaves. A high photosynthesis rate may cause flowering before the time that can lead to high productivity [127]. LR-motif is directly associated with the involvement in light responsiveness activity in different developmental stages in plant species [128–130]. These results suggest that identified LR-CAREs have a direct involvement in enhancing the rate of photosynthesis in sweet orange leaves.

The proper growth and development of plants mainly depend on phytohormones [131]. In this study, we also predicted some important hormone-responsive cis-elements such as ABRE involved in the abscisic acid responsiveness [132, 133], AuxRR-core associated with auxin responsiveness [125, 134], CGTCA-motif, GC motif related to anoxic specific inducibility [135], O2-site involved in zein metabolism regulation [125, 134], TCA-element involved in salicylic acid responsiveness), TGA-element associated with auxin-responsiveness [136, 137] and TGACG-motif. GARE motif, AuxRE, P box, and TATC box; Gibberellin-responsive elements play vital roles in seed germination, fruit senescence, flower development, leaf expansion, and shoot elongation [138]. We also found DRE (CARE involved in dehydration, low temperature, and salt stress), TC-rich repeats (associated with defense and stress response), MBS (dedicated to drought inducibility), and LTR (engaged in low-temperature response) those served as stress-responsive CAREs in several plant species [133, 139–141]. Other motifs like A box 1, AACA_motif, AT-rich element, AT-rich sequence, Box II-like sequence, Box III, HD Zip1, HD Zip3, MBSI, motif I, RY elements, and Unnamed_1 related to multiple biological functions were observed to be extremely shared by many predicted lectin genes in sweet orange. Also, we found a few unknown cis-elements in Cslectins. CAREs associated with the identified lectin gene in sweet orange may provide important clues for further detailed study of the regulation of plant growth, development, stress resistance, and defense mechanism against pathogens.

## 4.0 Conclusion

Globally, sweet orange fruit ranks in the second-highest position in terms of production rate. In this study, we employed bioinformatics approaches to identify and *in silico* characterize lectin genes in the sweet orange genome. We identified a total of 141 lectin genes within the whole sweet orange genome. Furthermore, we classified all identified Cslectin gene families into ten groups: 68 CsB-Lectin, 13 LysM, 4 CsChitin-Bind1, 1 CsLec-C, 3 CsGal-B, 1 CsCalreticulin, 3 CsJacalin, 13 CsPhloem, 11 CsGal-Lec, and 24 CsLectinlegB genes based on phylogenetic tree and typical conserved domain analysis. Analysis of gene structure (exon-intron numbers), conserved domain and motif composition revealed the highest similarity with the corresponding Arabidopsis lectin gene family. Furthermore, the GO analysis uncovered significant biological functions associated with the predicted lectin genes, such as defense against metabolic functions, and biotic and abiotic stresses. Sub-cellular localization showed an abundance of identified protein signals in the plasma membrane and extracellular region. We also constructed a regulatory network involving key TFs and the identified Cslectin genes. The predicted TFs and CAREs of Cslectin genes were demonstrated to be linked to the regulation of gene expression and plant growth. Consequently, our overall findings would pave the way for future wet-lab experiments involving the unraveled lectin genes in sweet oranges, which will elucidate their functional roles in growth, development, stress responses, defense against pathogen attacks, and enhanced productivity of sweet oranges. Additionally, information insight into this study will be beneficial for future breeding programs of this valuable fruit species targeting human health.

## Supporting information

S1 Data Full-length coding sequences of lectin gene superfamily families of *C. sinensis* plant species were used in this study. (TXT)

S2 Data Full-length genomic sequences of lectin gene superfamily families of *C. sinensis* plant species were used in this study. (TXT)

S3 Data Full-length protein sequences of lectin gene superfamily families of *C. sinensis* plant species were used in this study. (TXT)

S4 Data Full-length protein sequences of lectin gene superfamily families of *A. thaliana* and *C. sinensis* plant species were used for phylogenetic tree construction. (TXT)

S5 Data The detailed GO analysis of the identified lectin gene superfamily in *C. sinensis* was performed using the online tool Plant Transcription Factor Database PlantTFDB 4.0), (http://planttfdb.gao-lab.org/). (XLSX)

S6 Data Identified in a total of 278 unique TFs associated with the regulation of identified lectin gene superfamily in *C. sinensis* genome. (XLSX)

S7 Data The predicted *cis*-acting regulatory elements of the upstream promoter region (1.5 kb genomic sequences) of the lectin gene superfamily in *C. sinensis*. (XLSX)
